# Application of optical coherence tomography in cardiovascular diseases: bibliometric and meta-analysis

**DOI:** 10.3389/fcvm.2024.1414205

**Published:** 2024-07-09

**Authors:** Wenjing Lian, Cong Chen, Jie Wang, Jun Li, Chao Liu, Xueying Zhu

**Affiliations:** ^1^ Guang’anmen Hospital, China Academy of Chinese Medicine Sciences, Beijing, China; ^2^ Department of Anatomy, School of Traditional Chinese Medicine, Beijing University of Chinese Medicine, Beijing, China

**Keywords:** optical coherence tomography, cardiovascular diseases, bibliometric analysis, meta-analysis, percutaneous coronary intervention

## Abstract

**Significance:**

Since the advent of Optical Coherence Tomography (OCT) two decades ago, there has been substantial advancement in our understanding of intravascular biology. Identifying culprit lesion pathology through OCT could precipitate a paradigm shift in the treatment of patients with Acute Coronary Syndrome. Given the technical prowess of OCT in the realm of cardiology, bibliometric analysis can reveal trends and research focal points in the application of OCT for cardiovascular diseases. Concurrently, meta-analyses provide a more comprehensive evidentiary base, supporting the clinical efficacy of OCT-guided Percutaneous Coronary Intervention (PCI).

**Design:**

This study employs a dual approach of Bibliometric and Meta-analysis.

**Methods:**

Relevant literature from 2003 to 2023 was extracted from the Web of Science Core Collection (WoSCC) and analyzed using VOSviewer, CiteSpace, and R for publication patterns, countries, institutions, authors, and research hotspots. The study compares OCT-guided and coronary angiography-guided PCI in treating adult coronary artery disease through randomized controlled trials (RCTs) and observational studies. The study has been reported in the line with PRISMA and AMSTAR Guidelines.

**Results:**

Adhering to inclusion and exclusion criteria, 310 publications were incorporated, demonstrating a continual rise in annual output. Chinese researchers contributed the most studies, while American research wielded greater influence. Analysis of trends indicated that research on OCT and angiography-guided PCI has become a focal topic in recent cohort studies and RCTs. In 11 RCTs (*n* = 5,277), OCT-guided PCI was not significantly associated with a reduction in the risk of Major Adverse Cardiac Events (MACE) (Odds ratio 0.84, 95% CI 0.65–1.10), cardiac death (0.61, 0.36–1.02), all-cause death (0.7, 0.49–1.02), myocardial infarction (MI) (0.88, 0.69–1.13), target lesion revascularization (TLR) (0.94, 0.7–1.27), target vessel revascularization (TVR) (1.04, 0.76–1.43), or stent thrombosis (0.72, 0.38–1.38). However, in 7 observational studies (*n* = 4,514), OCT-guided PCI was associated with a reduced risk of MACE (0.66, 0.48–0.91) and TLR (0.39, 0.22–0.68).

**Conclusion:**

Our comprehensive review of OCT in cardiovascular disease literature from 2004 to 2023, encompassing country and institutional origins, authors, and publishing journals, suggests that OCT-guided PCI does not demonstrate significant clinical benefits in RCTs. Nevertheless, pooled results from observational studies indicate a reduction in MACE and TLR.

## 1 Introduction

Recent evidence suggests that MACE in chronic ischemic heart disease correlates more with the overall atherosclerotic burden than with specific flow-limiting luminal lesions (1–6). Traditional models simplistically link CAD complications to severe obstructions from narrow atherosclerotic plaques (7–10). However, this perspective is increasingly recognized as overly reductionist. Longitudinal studies on the natural progression of individual coronary plaques have revealed that even those lesions perceived as high-risk and potentially ischemia-inducing maintain stability over several years, seldom progressing to instability or resulting in MACE (11–16). The limitations of angiography in direct PCI, including inaccurate assessments of lesion morphology and the underlying mechanisms of STEMI, as well as suboptimal recognition of post-stent outcomes, underscore the necessity for a more holistic understanding of atherosclerosis within the entire arterial system (17).

OCT provides the highest resolution (1–15 μm) among current intravascular imaging technologies, enabling detailed exploration of microscopic vascular structures (18). In cardiovascular clinical applications, the significance of OCT encompasses: (1) Comprehensive plaque assessment: OCT provides detailed information about plaque size, type, and composition, aiding in understanding the total burden of atherosclerosis, not merely localized stenosis (19); (2) Vulnerable plaque identification: OCT can provide detailed views of potentially hazardous plaques by analyzing tissue characteristics, such as the size of the lipid core and the thickness of the fibrous cap (20); (3) Enhanced risk stratification: The detailed plaque and vascular information provided by OCT can help more accurately assess the risk of cardiovascular events, thus improving the accuracy of risk stratification (21); (4) Complementing traditional imaging techniques: By offering direct observation of vessel walls and plaques, OCT supplements the limitations of traditional imaging methods, providing a more comprehensive cardiovascular health assessment (22). Thus, OCT is not only a potent diagnostic tool but also adds a new dimension to the risk assessment and management of cardiovascular diseases. Its application highlights a deeper and more nuanced understanding of cardiac diseases, contributing to the refinement of existing risk stratification methods for greater precision.

We analyzed trends and applications of OCT in cardiovascular treatment over the past two decades using bibliometric techniques (23, 24). Our meta-analysis indicates OCT as a prominent focus in recent PCI trials. Previous studies comparing OCT-guided with angiography-guided PCI treatment in Meta-analyses have encountered several issues. Firstly, they did not include all significant related studies. Secondly, these meta-analyses did not separate observational studies from RCTs, a methodological rigor essential for enhancing the credibility of results. Therefore, we conducted a stringent Meta-analysis, differentiating RCTs from observational studies, aiming to provide more accurate and reliable evidence to guide clinical practice and future research directions.

## 2 Methods

### 2.1 Data sources and search strategy

**Figure 1:**
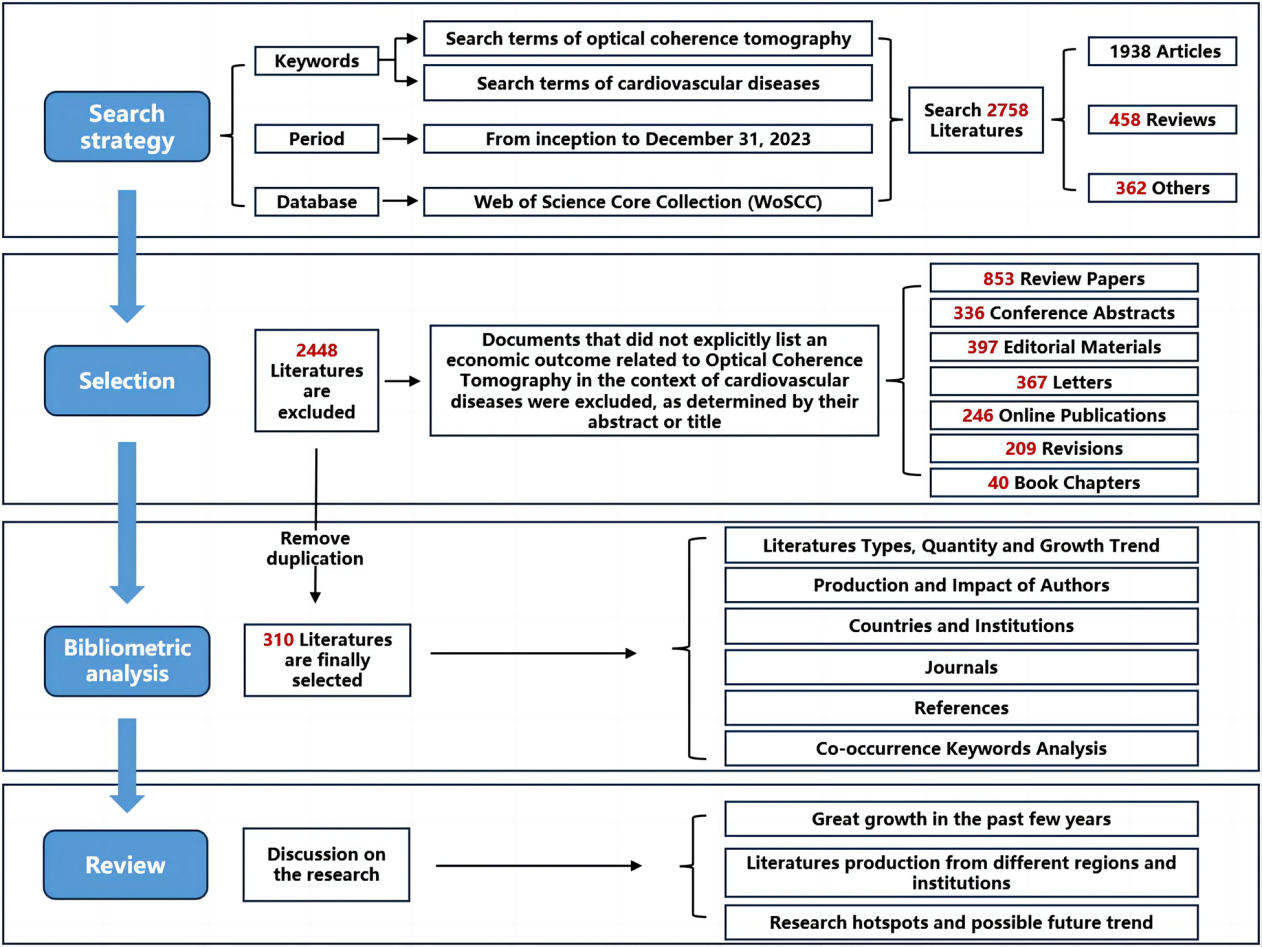
Document selection and flow chart of the research framework.

The Web of Science, esteemed for its extensive interdisciplinary coverage, comprehensive citation indexing, and rich analytical metrics, serves as an exemplary database for bibliometric analysis. This resource enables researchers to identify hotspots and trends within their respective fields. Our study utilized data retrieved from the WoSCC database concerning OCT and cardiovascular diseases for bibliometric analysis. To mitigate data variability due to updates, search activities, data extraction, and downloading were conducted on the same day. The types of literature studied were confined to articles and reviews. The search strategy, specific outcomes, and search terms are detailed in Figure 1 (refer to Supplementary eMethods S1). Overall, 2,758 literature sources were analyzed, with 310 articles ultimately included and downloaded in text format (complete records and referenced citations).

### 2.2 Data analysis and visualization

In this study, the bibliometrix package in R (version 4.3.2) was utilized to analyze major countries, active authors and institutions, contributing journals, and keyword trends (25). Additionally, CiteSpace (version 6.1), a Java-based freeware developed by Chen (26), was employed for clustering and burst analysis of keywords. Collectively, these two software programs facilitated visual analyses, offering deep insights into the advancements in OCT research within the cardiovascular field and uncovering research frontiers using extensive data.

### 2.3 Meta-analysis

This work has been reported in line with the PRISMA (Preferred Reporting Items for Systematic Reviews and Meta-Analyses) and AMSTAR (Assessing the methodological quality of systematic reviews) Guidelines (27, 28). A systematic review and meta-analysis were conducted on data from 11 RCTs and 7 observational studies. These 18 cohorts were identified through searches of electronic databases including PubMed, Cochrane Library, Embase, and Web of Science, employing a combination of text and MeSH headings in the search strategy (refer to Supplementary eMethods S2 and eTableS1). For this study, primary outcomes of interest were MACE, Cardiac death, and All-cause death, with secondary endpoints including Myocardial Infarction (MI), TVR, TLR, and stent thrombosis. All details regarding the search strategy, data extraction, and study selection are presented in the Supplementary (eMethods S3–S5).

### 2.4 Statistical analysis

The outcomes of interest were dichotomous variables, and rates of events with the total sample size were extracted for analysis. The Mantel-Haenszel method's random-effects model was employed to calculate Odds Ratio (OR) and their 95% Confidence Intervals (CI). For inter-study variance, Restricted Maximum Likelihood (REML) was used. An OR estimate and its corresponding 95% CI not including the vertical line at 1 (*p*-value < 0.05) was considered statistically significant. The extent of heterogeneity was approximated using the *I*^2^ test, with 0%–40% indicating negligible, 30%–60% moderate, 50%–90% substantial, and 75%–100% considerable heterogeneity. Given the limited number of studies included, a funnel plot for pre-specified publication bias analysis was deemed inappropriate.

### 2.5 Cardiovascular clinical research and patients involvement

Following the completion of our initial manuscript, we consulted a patient with cardiovascular disease and a frontline cardiovascular clinical scholar, both of whom suggested acceptance or implementation of PCI for coronary artery disease. The feedback received indicated that the certainty of the evidence presented in our study was highly useful for evaluating the efficacy of OCT-guided vs. angiography-guided PCI in the treatment of acute coronary syndrome (ACS).

## 3 Results

### 3.1 Bibliometric results

#### 3.1.1 Annual growth trends in publications

**Figure 2:**
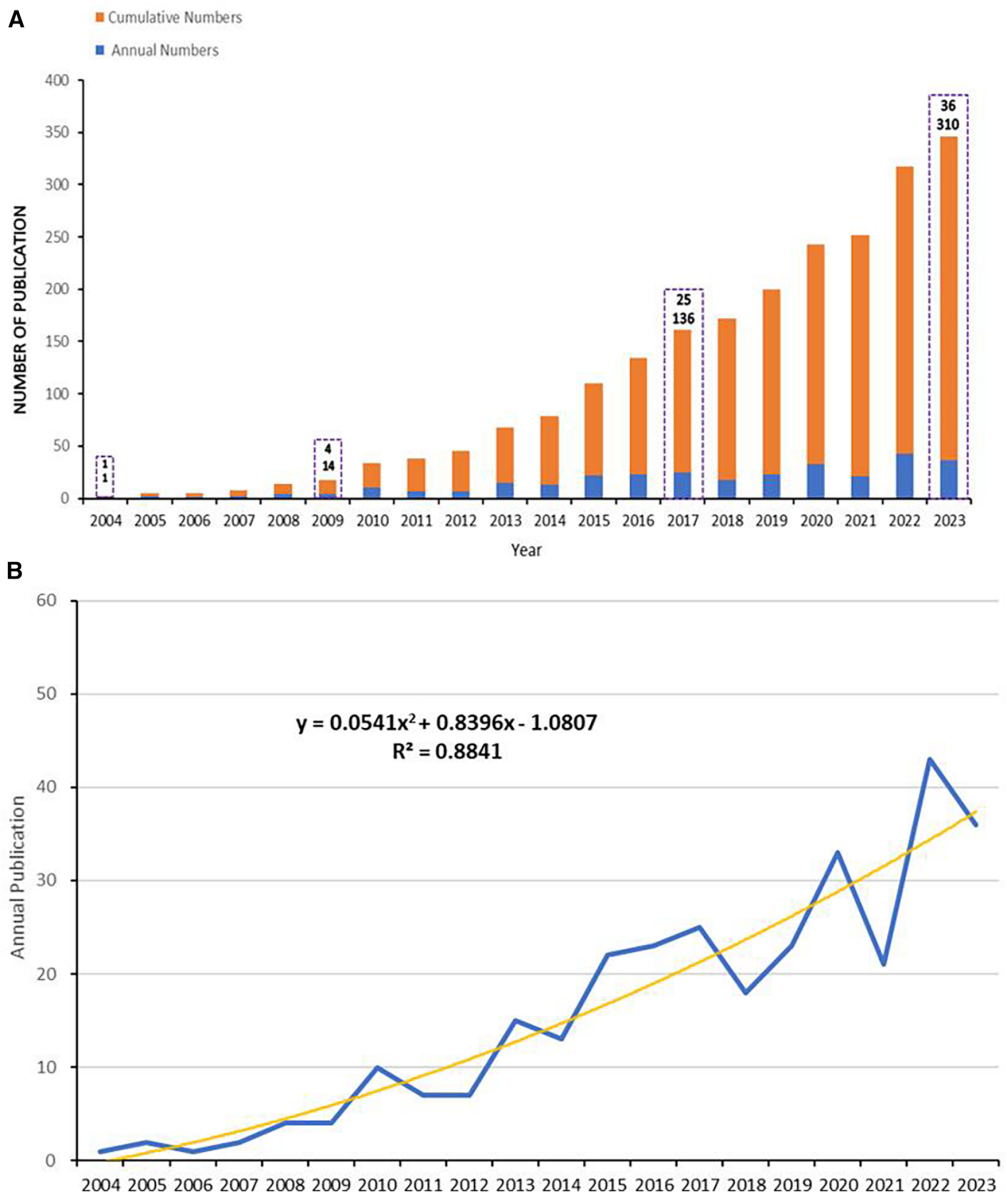
Annual publications, and fitting curves. (**A**) Cumulative (in orange-red) and annual (in blue) number of publications from 2004 to 2023. (**B**) The trend curve fitted according to the number of publications, depicted using polynomial fitting in Microsoft Excel 2019.

From 2003 to 2023, a total of 2,758 papers were retrieved from the WoSCC database. After eliminating duplicates and other types of literature, 310 articles were ultimately included for analysis, comprising 246 articles and 64 reviews. Figure 2A displays the annual statistics of publications in this field, revealing a trend in three distinct phases: (1) From 2004 to 2012, the annual publication count did not exceed 10 papers; (2) From 2013 to 2019, the number of annual publications remained relatively stable; (3) A notable increase in publication volume was observed from 2020 to 2023, with a significant spike exceeding 40 papers in 2022. By fitting the data to construct a publication trend, results indicate a high correlation between the annual number of publications and the years (*y* = 0.0541x ^2 + 0.8396x − 1.0807, R^2 = 0.8841) (Figure 2B). The publication trend suggests that by 2024, over 400 articles on this topic are projected to be published, signifying an increasing scholarly focus on this field over time.

**Figure 3:**
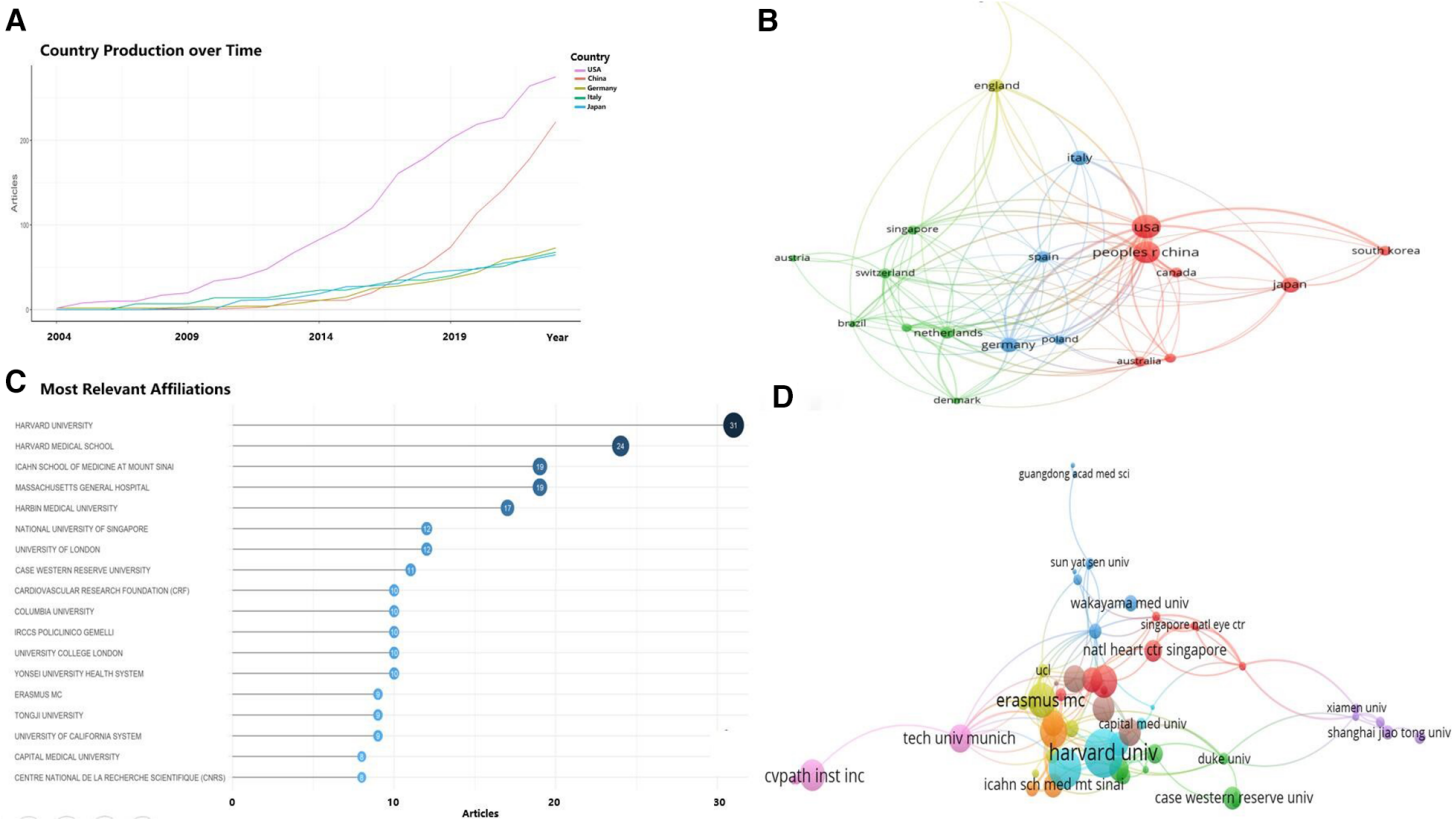
Analysis of countries/regions engaged in OCT research. (**A**) Top 5 countries with the largest number of publications over time; (**B**) country cooperation network; (**C**) top 18 institutions by number of publications; (**D**) institutional collaboration-network.

In this field, the top 10 countries accounted for over 80% of the total publication output compared to all other countries combined. Statistically, the five countries and regions with the most published articles were China (77 articles), the United States (68 articles), Germany, Japan, and Italy (Table 1). In terms of the growth rate in the number of publications (Figure 3A), the United States consistently maintained a high output, slightly outperforming China, while Germany, Italy, and Japan showed relatively stable production levels. Moreover, among the top 20 countries for corresponding authors, those with the highest proportions of multiple countries publication (MCP) relative to their total publication output were Canada, the United States, Italy, the United Kingdom, and China. Although the U.S. had the most MCPs (23 articles), it did not rank first in MCP ratio. While China had the highest total number of articles, it had fewer publications in collaboration with other countries (22 articles), thus a lower MCP Ratio (Table 1). Among the limited international collaborations from China, those with the United States were the most frequent (Figure 3B).

**Table 1:** Corresponding author's countries.

Rank	Country	Articles	SCP	MCP	MCP Ratio
1	China	77	55	22	0.286
2	Usa	68	45	23	0.338
3	Germany	20	15	5	0.25
4	Japan	20	20	0	0
5	Italy	18	12	6	0.333
6	Canada	12	6	6	0.5
7	United Kingdom	10	7	3	0.3
8	Spain	9	7	2	0.222
9	France	8	6	2	0.25
10	Korea	7	4	3	0.429

MCP, multiple countries publication; SCP, single countries publication.

These articles were authored by 659 institutions, among which 21 institutions published at least 5 articles each. The top 10 institutions alone authored 176 articles, accounting for 56.8% of the total (Figure 3C). The institutions with the highest number of publications included Harvard University, Harvard Medical School, Harbin Medical University, Icahn School of Medicine, Massachusetts General Hospital, National University of Singapore, University College London, Case Western Reserve University, and Columbia University, all with over 10 articles each. Chinese institutions such as Harbin Medical University, Tongji University, and Capital Medical University each produced more than 8 articles. As depicted in Figure 3D, institutional collaboration was more extensive than inter-country cooperation, with Harvard University and Harbin Medical University engaging in significant collaborations with numerous universities and research centers in China, as well as institutions in the UK, the US, and other countries.

#### 3.1.2 Author analysis

**Figure 4:**
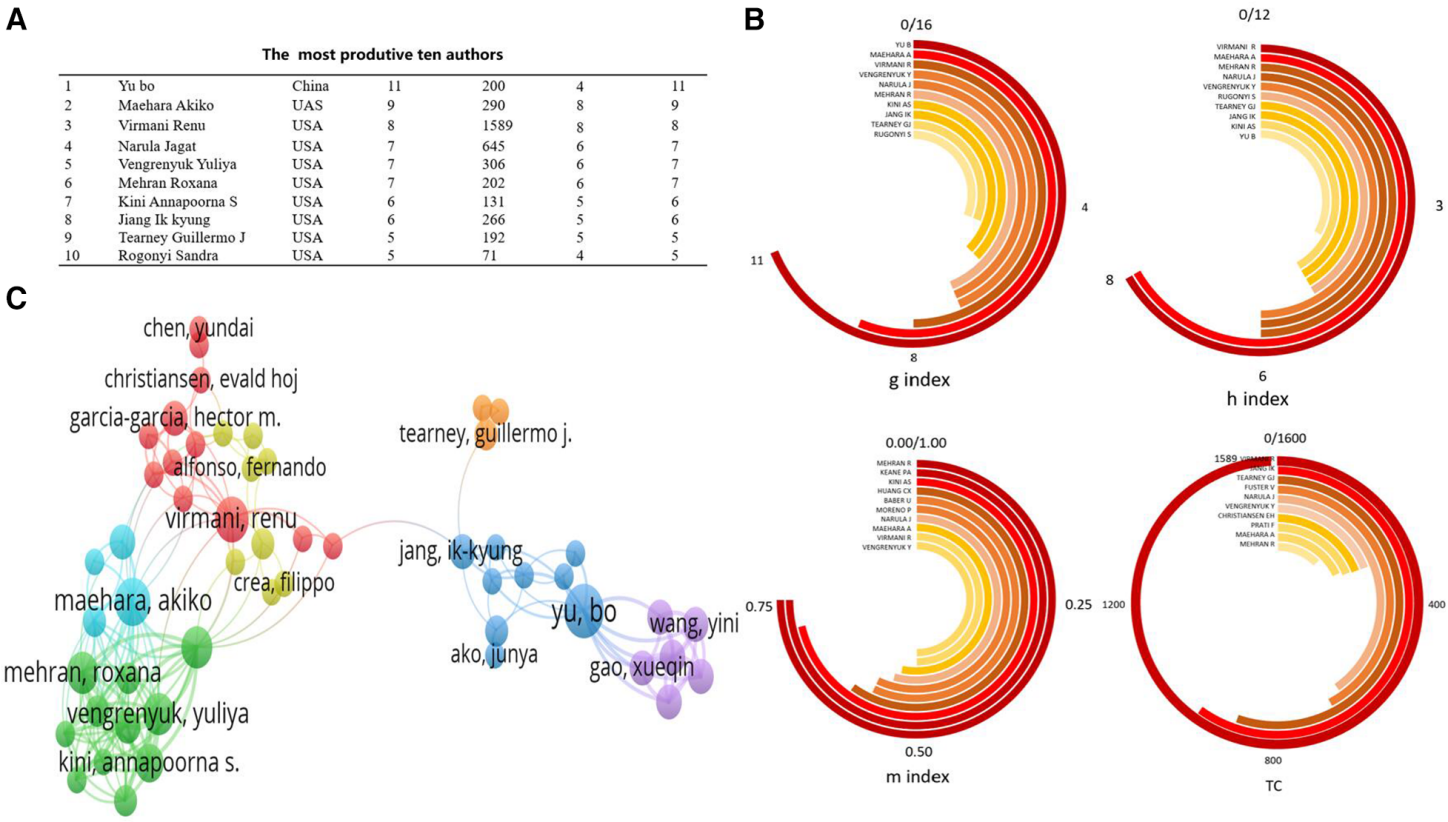
Analysis of authors engaged in OCT research. (**A**) the most produtive ten authors; (**B**) Under Various Indices, the Top 10 Most Productive Authors; (**C**) author's cooperation network.

Figure 4A, created using VOSviewer software, visualizes the author collaboration network in OCT research within the cardiovascular field. The minimum criterion for an author's inclusion was set at 10 publications, encompassing nearly 2000 contributing authors. Among the top 10 most productive authors, Professor Mehran Roxana possessed the highest m-INDEX; Professor Yu Bo boasted the greatest G-index; and Professor Virmani Renu held the highest h-index and total citations (TC) (Figures 4A-B).

Figure 4C presents the network map of co-cited authors, where higher weightage of a co-cited author corresponds to larger labels and circles in the visualization. In the field of OCT research, prominent figures like Professor Yu Bo from China, and Professor Maehara Akiko and Professor Virmani Renu from the United States, hold significant influence and citation weight. Among the authors with the highest publication volumes, Professor Yu Bo is the only one from China, while the others are predominantly from the United States. Many of these authors have collaborated on publications in journals such as the “New England Journal of Medicine” and “JACC Cardiovascular Imaging” (29, 30). This indicates close collaboration among authors within this field.

#### 3.1.3 Analysis of journals

**Figure 5:**
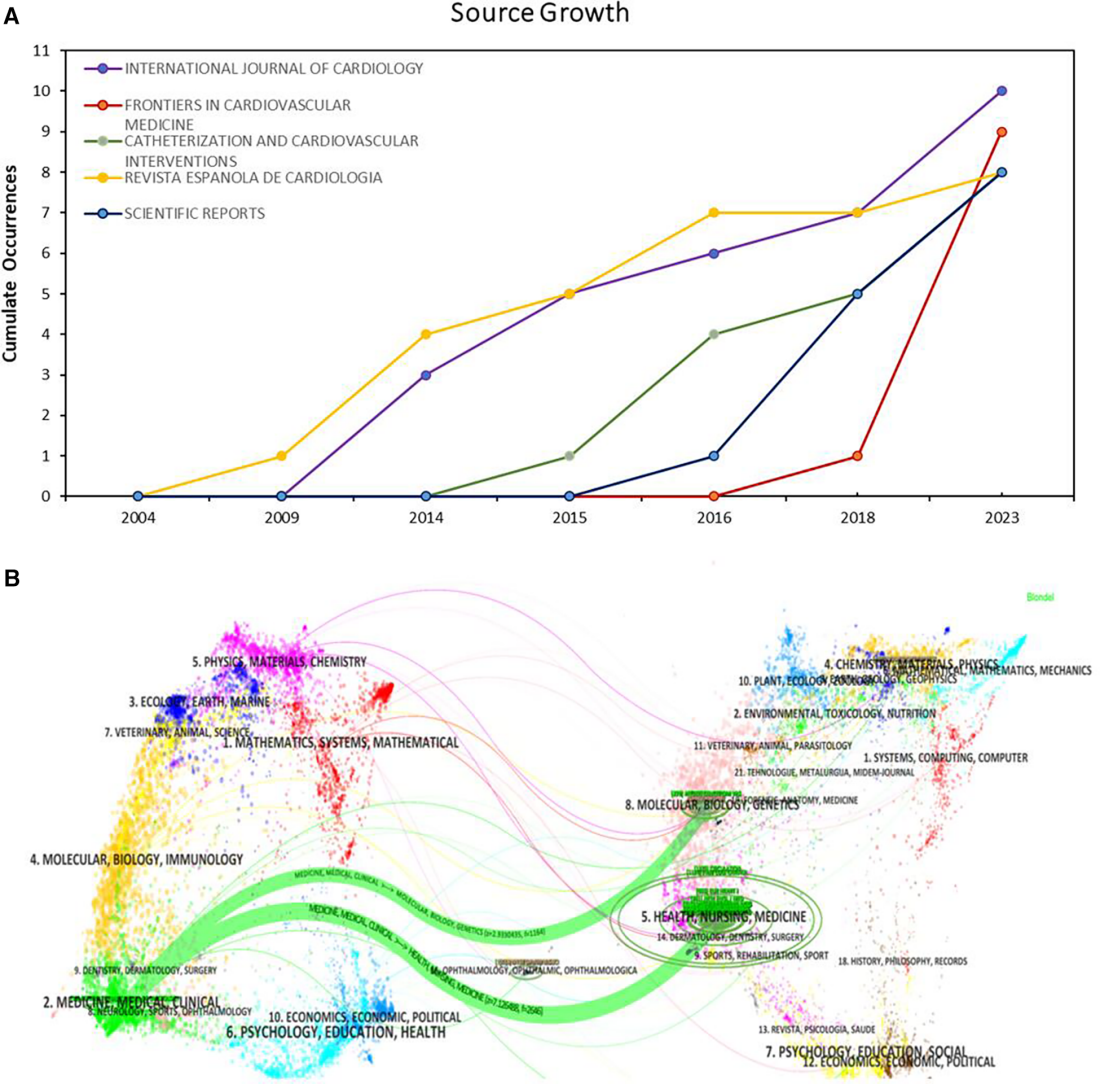
Journal distribution. (**A**) Growth Trends of the Top 5 Productive Journals; (**B**) dual map overlay for journals.

According to our analysis, 179 journals published papers related to OCT and cardiovascular diseases. The top 5 most productive journals — “International Journal of Cardiology”, “Frontiers in Cardiovascular Medicine”, “Catheterization and Cardiovascular Interventions”, “Revista Espanola de Cardiologia”, and “Scientific Reports” — showed notable publication numbers and growth trends, as depicted in Figure 5A. Figure 5B illustrates the journal's thematic distribution through a dual-map overlay, with citing journals positioned on the left and cited journals on the right of the map. The labels represent journals covering specific themes, and colored lines trace the reference pathways. Two distinct citation pathways are evident. Two green citation paths indicate that studies from medical/clinical/surgical journals are often cited by those in molecular physiology/medical/clinical journals.

#### 3.1.4 Citation analysis

**Figure 6:**
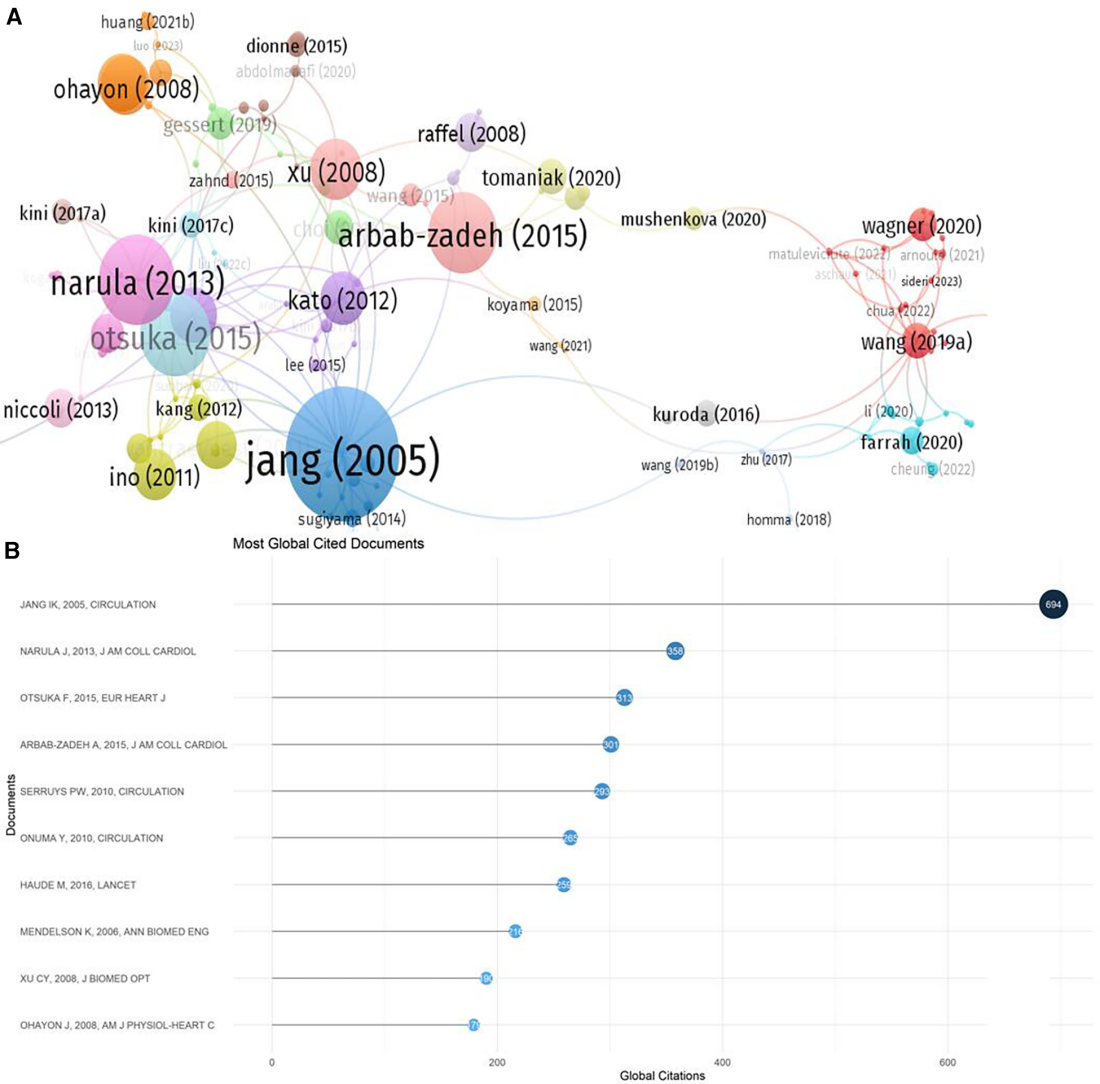
Journal distribution. (**A**) Co-citation network; (**B**) top 10 most cited papers.

The results of the citation analysis are presented in Figure 6 and Table 2. Among the top 10 most cited articles, 6 are clinical trial studies, with one published in “The Lancet" (37), three in “Circulation" (31, 35, 36), and other high-impact journals. Three reviews discussed the application of OCT technology in detecting atherosclerosis in clinical practice. The 2005 randomized controlled trial by Professor Ik-Kyung Jang, “*in vivo* Characterization of Coronary Atherosclerotic Plaque by Use of Optical Coherence Tomography,” ranks first with 694 citations.

**Table 2:** Top 10 most cited publications.

Rank	Representative author	Title	Key points	Journal	Citations (2023)	Year	Type	IF (2022)
1	Ik-Kyung Jang (31)	*in vivo* Characterization of Coronary Atherosclerotic Plaque by Use of Optical Coherence Tomography	This is the first study to compare detailed *in vivo* plaque morphology in patients with different clinical presentations.	Circulation	694	2005	RCT	37.8
2	Jagat Narula (32)	Histopathologic Characteristics of Atherosclerotic Coronary Disease and Implications of the Findings for the Invasive and Noninvasive Detection of Vulnerable Plaques	It defines the histomorphological characteristics of vulnerable plaques, helping to identify such plaques in patients at high risk of acute coronary events.	Journal of the American College of Cardiology	358	2013	RCT	24.0
3	Fumiyuki Otsuka (33)	Neoatherosclerosis: overview of histopathologic findings and implications for intravascular imaging assessment	It explaines the introduction of OCT has facilitated the detection of new atherosclerosis in clinical practice OCT.	Eur Heart J	313	2015	Review	39.3
4	Armin Arbab-Zadeh (34)	The Myth of the “Vulnerable Plaque”: Transitioning From a Focus on Individual Lesions to Atherosclerotic Disease Burden for Coronary Artery Disease Risk Assessment	It supports the multifaceted hypothesis of the natural course of atherosclerotic plaque rupture is summarized.	J Am Coll Cardiol	301	2015	Review	24.0
5	Patrick W Serruys (35)	Evaluation of the Second Generation of a Bioresorbable Everolimus Drug-Eluting Vascular Scaffold for Treatment of *de novo* Coronary Artery Stenosis	It is This first-in-humans trial provides a seminal observation on a small number of patients with a short duration of follow-up. One of the clinical studies looked at the Second Generation of a Bioresorbable Everolimus Drug-Eluting Vascular Scaffold for Treatment of De with OCT Novo Coronary Artery Stenosis.	Circulation	293	2010	RCT	37.8
6	Yoshinobu Onuma (36)	Intracoronary Optical Coherence Tomography and Histology at 1 Month and 2, 3, and 4 Years After Implantation of Everolimus-Eluting Bioresorbable Vascular Scaffolds in a Porcine Coronary Artery Model	It reports OCT findings with corresponding histology in the porcine coronary artery model immediately after and at 28 days and 2, 3, and 4 years after BVS implantation.	Circulation	265	2010	RCT	37.8
7	Michael Haude (37)	Safety and performance of the second-generation drug-eluting absorbable metal scaffold in patients with de-novo coronary artery lesions (BIOSOLVE-II): 6 month results of a prospective, multicentre, non-randomised, first-in-man trial	It evaluates the safety and performance of a new second-generation drug-eluting absorbable metal stent (DREAMS 2G) in patients with coronary neoplasia.	Lancet	259	2016	RCT	168.9
8	Karen Mendelson (38)	Heart Valve Tissue Engineering: Concepts, Approaches, Progress, and Challenges	It evaluates optical coherence tomography can be used to evaluate tissue remodeling for cardiac valve tissue engineering applications.	Ann Biomed Eng	216	2006	Review	3.8
9	Chenyang Xu (39)	Characterization of atherosclerosis plaques by measuring both backscattering and attenuation coefficients in optical coherence tomography	It addresses the fundamental issues that underlie the tissue characterization of OCT images obtained from coronary arteries. It not only explains the origins of many qualitative OCT features, but also shows that combination of backscattering and attenuation coefficient measurements can be used for contrast enhancing and better tissue characterization.	J Biomed Opt	190	2008	RCT	3.5
10	Jacques Ohayon (40)	Necrotic core thickness and positive arterial remodeling index: emergent biomechanical factors for evaluating the risk of plaque rupture	It demonstrats that plaque instability should not be considered as a result of fiber cap thickness alone, but rather as a combination of plaque thickness, necrotic core thickness, and arterial remodeling index.	Am J Physiol-Heart C	179	2008	RCT	4.8

#### 3.1.5 Co-occurrence analysis

**Figure 7:**
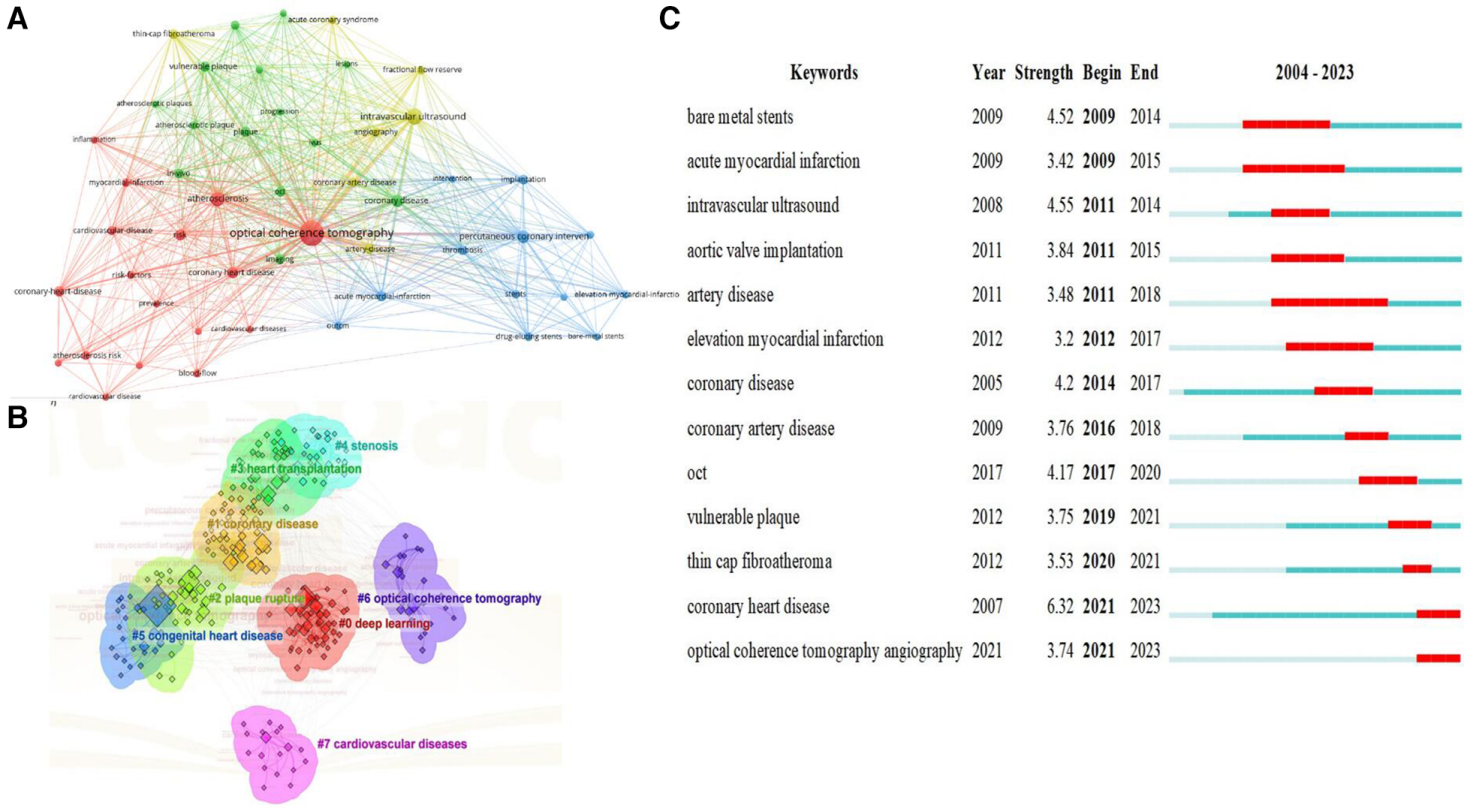
Visualized analysis of keywords and literature related to OCT and cardiovascular diseases. (**A**) Co-occurrence network of terms in 310 publications; nodes represent keywords (top 50), and lines denote co-occurrence relationships; (**B**) keyword clustering analysis; (**C**) the burst strength and duration of the top 13 keywords with the strongest citation bursts.

In the study of the structure of scientific knowledge, keyword co-occurrence analysis is an effective bibliometric method to grasp current hotspots. We analyzed the co-occurrence of keywords in the field and the top 50 keywords (Figure 7A), centering around OCT. Figure 7B employs a log-likelihood ratio analysis to generate eight clusters, including: coronary artery disease, deep learning, coronary stenosis, heart transplantation, plaque rupture, OCT, congenital heart disease, and cardiovascular diseases. Burst analysis of keywords was also conducted, revealing overall trends in OCT research in the cardiovascular field, encompassing topics like bare metal stents, acute myocardial infarction, intravascular ultrasound, aortic valve implantation, artery disease, elevation myocardial infarction, coronary disease, and coronary artery disease (Figure 7C).

#### 3.1.6 Changes in trends of research in the recent years

**Figure 8:**
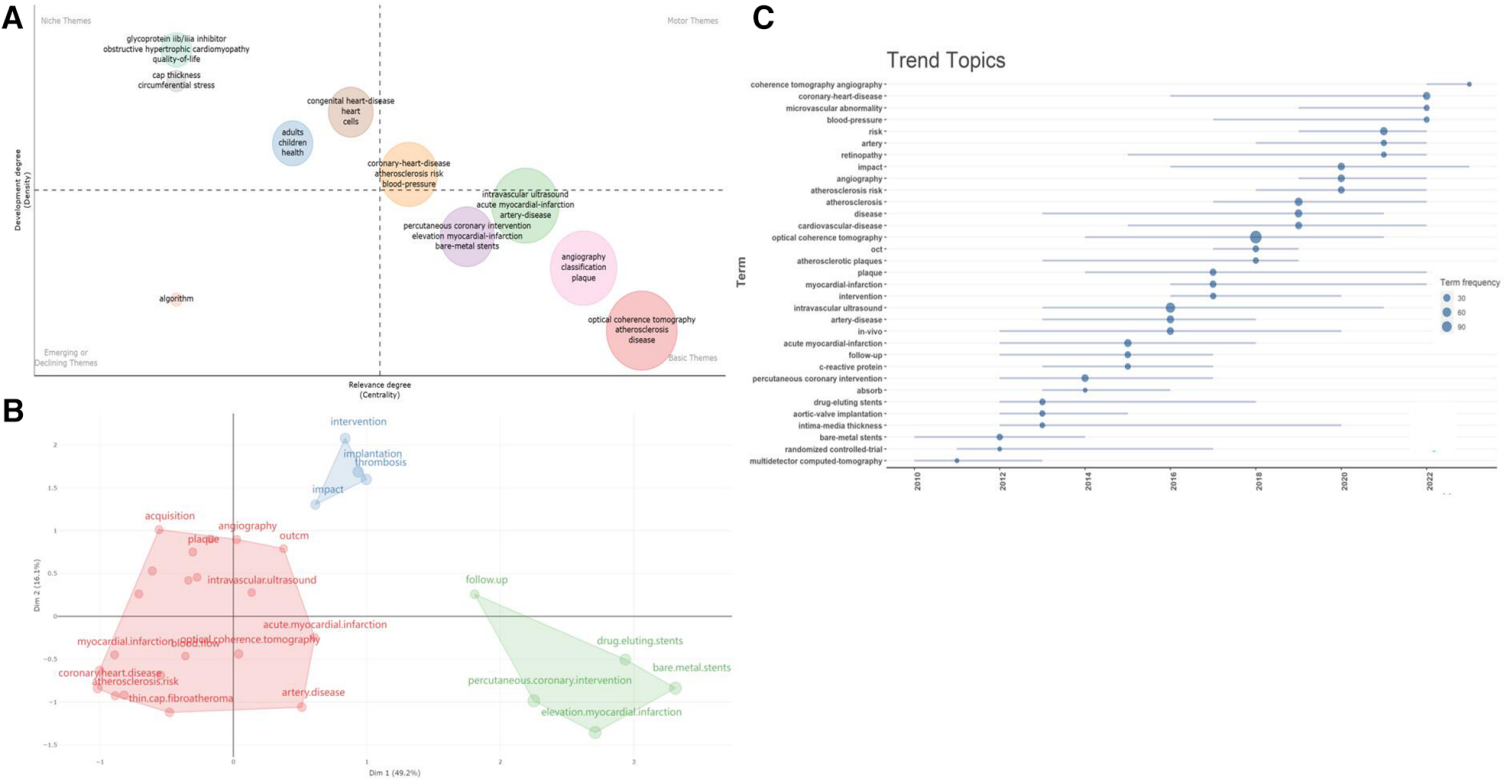
Analysis of research directions. (**A**) Thematic analysis related to cardiovascular diseases and OCT. The horizontal and vertical axes represent centrality and density, respectively. The first quadrant represents mature themes, the second quadrant is less significant to the current field, the third quadrant possibly represents emerging or fading themes, and the fourth quadrant is fundamental but less significant themes; (**B**) conceptual structure map of Keyword Plus; (**C**) timeline of research dynamics in the field of OCT and cardiovascular diseases.

The thematic word analysis method was employed to explore the core issues in OCT research within the cardiovascular field. Figure 8A indicates that well-developed themes focus on atherosclerotic diseases, blood pressure, stent implantation, and plaque characteristics. The impact of surgery, post-operative care, and survival on disease treatment and prognosis are also noteworthy. Emerging research in areas such as molecular biology and cell biology is also beginning to emerge. Researchers are focusing on the roles and potential molecular mechanisms of “inflammation, oxidative stress, mitochondria, cytokines, and metabolism” in disease development.

Moreover, using multidimensional scaling, we categorized the most frequently occurring keywords and generated a conceptual structure map, resulting in three clusters (Figure 8B). Current research continues to focus on clinical manifestations, diagnosis, interventions, and prognosis of diseases like “coronary stenosis, acute myocardial infarction, atherosclerosis” (red cluster), as well as exploring pathogenic mechanisms and intervention methods related to diseases, such as “interventional methods, post-stent thrombosis formation, and potential impacts of PCI” (blue-green cluster).

Additionally, we visualized the temporal trends of keywords (Figure 8C). In the past five years, new trends in the field include coherence tomographic vascular scanning technology, coronary heart disease, microvascular lesions, vascular pressure, retinal arteriolar abnormalities, atherosclerotic risk, as well as the etiology, pathomechanisms, and clinical outcomes of cardiovascular diseases, all of which are worthy areas for continued exploration. The thematic word analysis method was employed to explore the core issues in OCT research within the cardiovascular field. Figure 8A indicates that well-developed themes focus on atherosclerotic diseases, blood pressure, stent implantation, and plaque characteristics. The impact of surgery, post-operative care, and survival on disease treatment and prognosis are also noteworthy. Emerging research in areas such as molecular biology and cell biology is also beginning to emerge. Researchers are focusing on the roles and potential molecular mechanisms of “inflammation, oxidative stress, mitochondria, cytokines, and metabolism” in disease development.

Moreover, using multidimensional scaling, we categorized the most frequently occurring keywords and generated a conceptual structure map, resulting in three clusters (Figure 8B). Current research continues to focus on clinical manifestations, diagnosis, interventions, and prognosis of diseases like “coronary stenosis, acute myocardial infarction, atherosclerosis” (red cluster), as well as exploring pathogenic mechanisms and intervention methods related to diseases, such as “interventional methods, post-stent thrombosis formation, and potential impacts of PCI” (blue-green cluster).

Additionally, we visualized the temporal trends of keywords (Figure 8C). In the past five years, new trends in the field include coherence tomographic vascular scanning technology, coronary heart disease, microvascular lesions, vascular pressure, retinal arteriolar abnormalities, atherosclerotic risk, as well as the etiology, pathomechanisms, and clinical outcomes of cardiovascular diseases, all of which are worthy areas for continued exploration.

### 3.2 Meta results

#### 3.2.1 Description of included trials

**Figure 9:**
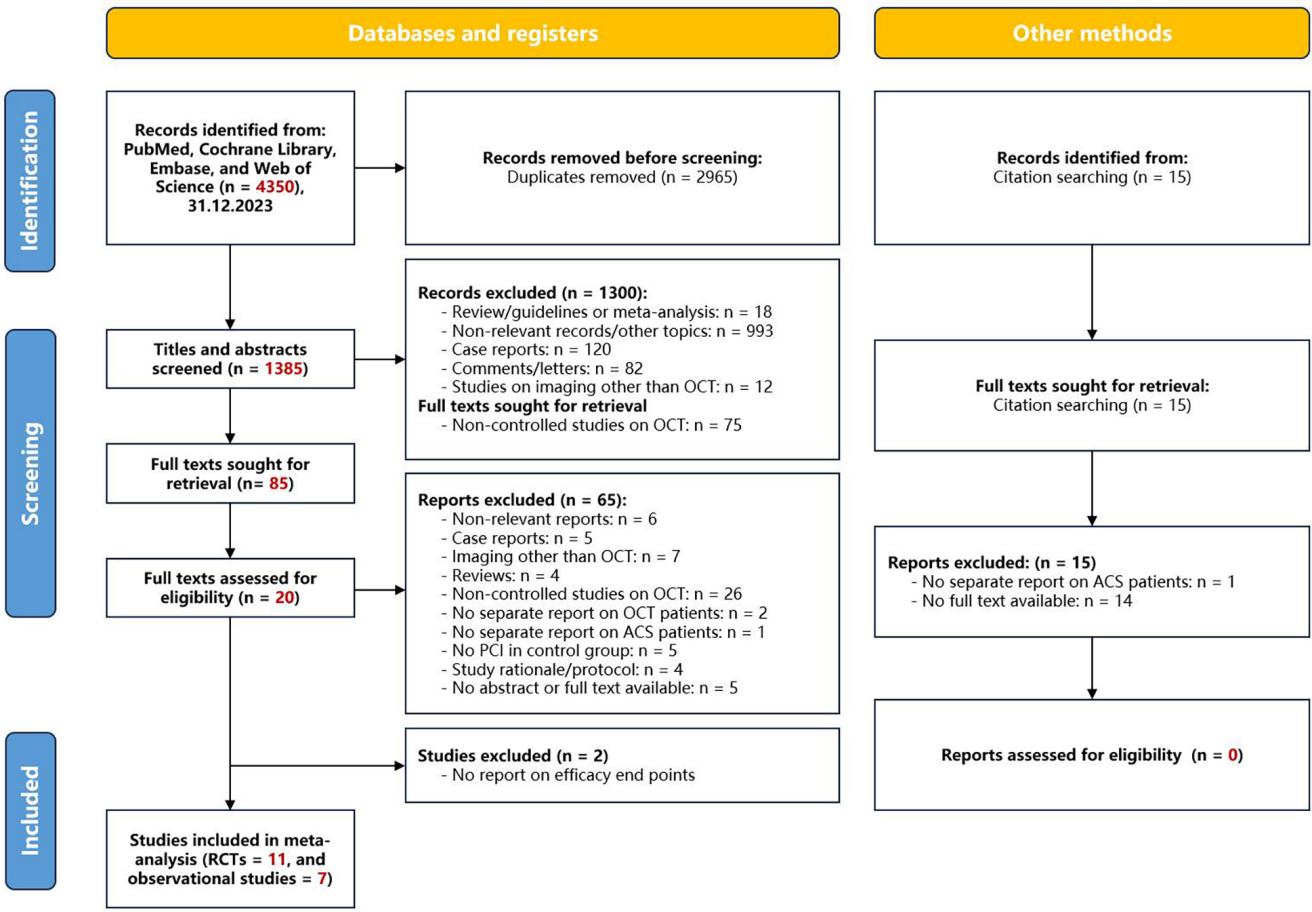
Flowchart of study selection. OCT, optical coherence tomography; PCI, percutaneous coronary intervention; ACS, acute coronary syndrome.

Of 4,350 citations, we reviewed 1,385 after removal of duplicates. We excluded an additional 1,367 studies on the basis of the title and abstract level screening and *a priori* selection criteria (Figure 9). Finally, we included 11 RCTs (*n* = 5,277) (29, 41–50), and 7 observational studies (*n* = 4,514) (51–58).

During the quality assessment process, a thorough evaluation of the methodological rigor of each study played a crucial role in enhancing the credibility of the results. Our bias risk assessment revealed that 36% (4 out of 11) of the trials raised some concerns regarding the randomization process, and 43% (3 out of 7) of the observational studies exhibited lower evidence quality regarding outcomes (refer to Supplementary eFigure S1 and eTable S2).

#### 3.2.2 Patient-level baseline characteristics and procedural data

The 11 articles included in this study collectively encompassed 5,277 patients with coronary artery lesions. Table 3 summarizes the baseline characteristics. The median age ranged from 54.5 to 69 years, with 77.2% being male. Cardiovascular risk factor analysis indicated that 31.5% of the patients had diabetes, 63.1% had dyslipidemia, and 69.0% had hypertension, with 31.7% being current or former smokers. STEMI was the predominant type of ACS, followed by NSTEMI and unstable angina. All patients underwent invasive treatment. A total of 2,653 patients received OCT-guided therapy, and 2,640 patients underwent angiography-guided PCI. Stent implantation was the primary strategy for vascular revascularization. The follow-up period ranged from 3 to 25 months. The characteristics of the observational studies are available in Supplementary eTable S3.

**Table 3:** Baseline demographics of trials and populations included in meta-analysis.

Study characteristics						Baseline characteristics of patients included (OCT group/Angiography group)							Type of patients and management (OCT group/Angiography group)		
Author Year	Location	Time Period Under Observation	Study type	Comparator treatment	Follow-up period (median)	Patients(Included in the analysis)	Age, median	Male patients	Hypertension	Diabetes	Smoking	Dyslipidemia	Unstable Angina	NSTEMI	STEMI
Ali 2023 (29)	Multinational	2018–2020	RCT(NCT03507777)	Angiography	12 months	1233/1254	65.5 (10.5)/65.7 (10.3)	968 (78.5)/956 (76.2)	880 (71.4)/928 (74.0)	523 (42.4)/521 (41.5)	242 (19.6)/247 (19.7)	808 (65.5)/860 (68.6)	355 (28.8)/331 (26.4)	304 (24.7)/299 (23.8)	68 (5.5)/73 (5.8)
Holm 2023 (44)	Multinational	2017–2022	RCT (NCT03171311)	Angiography	2 years	600/601	66.4 (10.5)/66.2 (9.9)	473 (78.8)/475 (79)	422 (70.3)/448 (74.5)	103 (17.2)/97 (16.1)	305 (50.8)/290 (48.3)	456 (76.0)/471 (78.4)	53 (8.8)/58 (9.7)	79 (13.2)/78 (13.0)	138 (23.0)/144 (24.0)
Jia 2022 (41)	China	2017–2019	RCT(NCT03571269)	Angiography	369 days	112/114	54.5 (11.2)/56.4 (10.4)	89 (79.5)/91 (79.8)	47 (42)/45 (39.5)	29 (25.9)/19 (16.7)	64 (57.1)/73 (64.0)	–	0/0	0/0	112 (100)/114 (100)
Ali 2021 (47)	Multinational	–	RCT(NCT02471586)	Angiography	6 months	153/142	66 (59–72) /67 (56–75)	106 (69)/104 (73)	120 (78)/104 (73)	51 (33)/40 (28)	26 (17)/40 (28)	112 (73)/110 (77)	–	–	50 (33)/51 (36)
Onuma 2020 (45)	Japan 9	2017–2018	RCT (NCT 0297248)	Angiography	6 months	55/50	68.9 (10.2)/69 (11.6)	44 (79)/40 (74)	43 (76.8)/40 (74.1)	29 (51.8)/25 (46.3)	13 (23.2)/10 (18.5)	48 (85.7)/ 46 (85.2)	4 (7.1)/2 (3.7)	1 (1.8)/1 (1.9)	–
Ueki 2020 (50)	Multinational	2016–2017	RCT(NCT02683356)	Angiography	6 months	19/19	63.3 (12.7)/62.9 (9.1)	15 (78)/15 (78)	7 (37)/11 (58)	4 (21)/4 (21)	7 (37)/6 (32)	13 (68)/ 12 (63)	4 (40)/1 (20)	4 (40)/2 (40)	2 (20)/2 (40)
Kala 2017 (46)	Czech Republic	2011–2012	RCT(NCT00888758)	Angiography	4.5months	105/96	57/59	87 (83)/83 (87)	53 (50)/50 (52)	18 (17)/25 (26)	67 (64)/57 (59)	–	–	–	105 (100)/96 (100)
Ali 2016 (49)	Multinational	2015–2016	RCT(NCT02471586)	Angiography	6 months	158/146	66 (59–72)/67 (56–75	109 (68)/107 (73)	124 (78)/109 (75)	52 (33)/42 (29)	28 (18)/35 (24)	115 (73)/112 (77)	25 (16)/27 (18)	20 (13)/24 (16)	6 (4)/4 (3)
Meneveau 2016 (42)	France	2013–2015	RCT(NCT01743274)	Angiography	6 months	120/120	60.8 (11.5)/60.2 (11.3)	95 (79.2)/91 (75.8)	67 (55.8)/50 (41.7)	26 (21.7)/19 (15.8)	47 (39.2)/51 (42.5)	59 (49.2)/56 (46.7)	10 (8.3)/9 (7.5)	110 (91.7)/111 (92.5)	–
Kim 2015 (48)	Korea	2011–2012	RCT(NCT01869842)	Angiography	6 months	58/59	58.8 (10.8)/61.6 (9.7)	39 (78)/37 (72)	27 (54.0)/25 (49.0)	16 (32.0)/16 (31.4)	16 (32.0)/15 (29.4)	33 (66)/ 37 (72.5)	–	–	–
Antonsen 2015 (43)	Denmark	2011–2013	RCT(NCT02272283)	Angiography	6 months	40/45	61.8 (9.4)/62.6 (11.0)	36 (72)/34 (68)	28 (56)/28 (56)	8 (16)/5 (10)	23 (46)/18 (36)	–	0/0	50 (100)/50 (100)	0/0

NSTEMI, non-ST-elevation myocardial Infarction; OCT, optical coherence tomography; RCT, randomized controlled trial; STEMI, ST-elevation myocardial infarction.

#### 3.2.3 MACE

**Figure 10:**
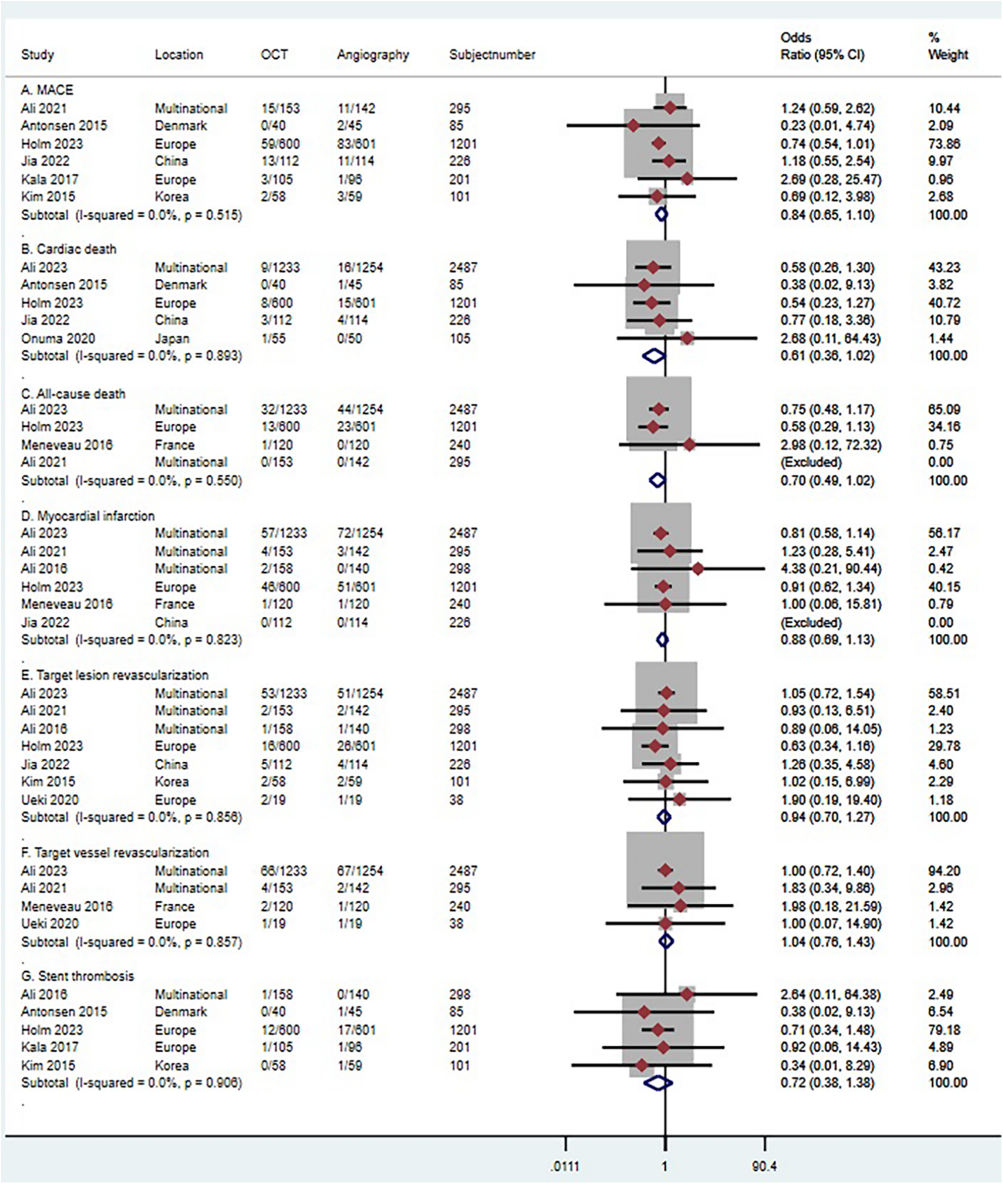
Forest plot comparing OCT guided with coronary angiography guided PCI in RCT studies. Data obtained from RCTs using fixed effect meta-analysis and expressed as OR. (**A**) MACE; (**B**) cardiac death; (**C**) all-cause death; (**D**) myocardial infarction; (**E**) target lesion revascularization (**F**) target vessel revascularization; (**G**) stent thrombosis. CI, confidence interval; OCT, optical coherence tomography; PCI, Percutaneous coronary intervention.

**Figure 11:**
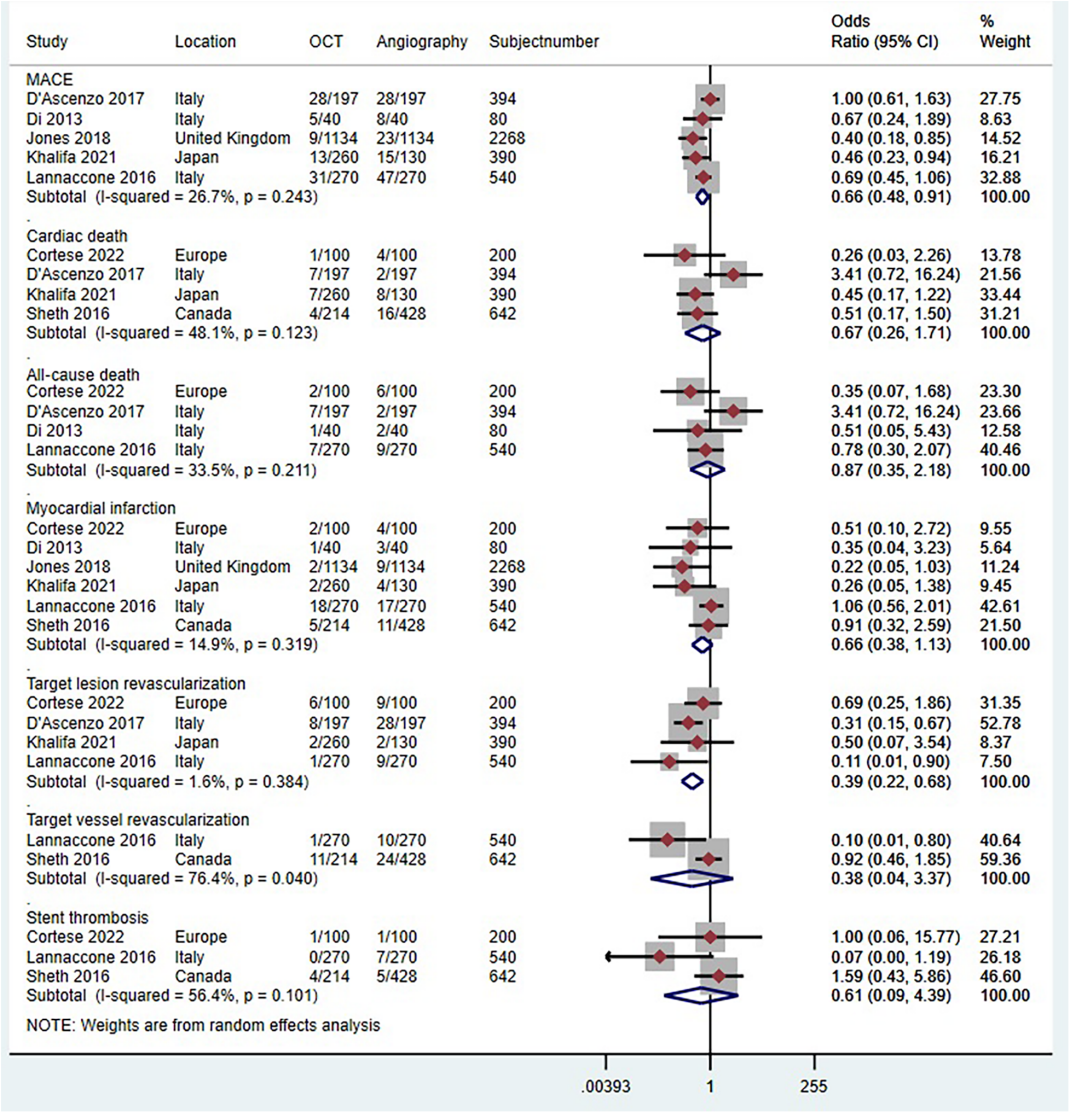
Forest plot comparing OCT guided with coronary angiography guided PCI for all-cause death. Data obtained from RCTs using fixed effect meta-analysis and expressed as OR. CI, confidence interval; OCT, optical coherence tomography; PCI, percutaneous coronary intervention.

Six trials (*n* = 2,109) reported MACE (41, 43, 44, 46–48). Compared with coronary angiography, OCT-guided PCI was not associated with a significant reduction in MACE (OR 0.84, 95% CI 0.65 to 1.10; *p* = 0.515, *I^2^*^ ^= 0%, non-relevant heterogeneity, high certainty, see Figure 10A). However, the observational studies, comprising 5 studies with 3,674 patients, painted a different picture (51, 52, 54–56). In contrast to coronary angiography, OCT-guided PCI showed a reduction in the risk of MACE (OR 0.66, 95% CI 0.48–0.91; *p* = 0.243, *I^2^*^ ^= 26.7%, non-relevant heterogeneity, moderate certainty, see Figure 11A).

#### 3.2.4 Cardiac death

Five trials (*n* = 4,104) reported cardiac death (29, 41, 43–45). Compared with coronary angiography, OCT-guided PCI was not associated with a significant reduction in cardiac death (OR 0.61, 95% CI 0.36–1.02; *p *= 0.893, *I^2^*^ ^= 0%, non-relevant heterogeneity, high certainty, see Figure 10B). Observational studies (51–53, 57), which included 4 studies with 1,626 patients, also demonstrated that OCT-guided PCI was not significantly associated with a reduction in the risk of cardiac death when compared to coronary angiography (OR 0.67, 95% CI 0.27–1.71; *p* = 0. 123, *I^2^*^ ^= 48.1%, indicating moderate heterogeneity and moderate certainty, as shown in Figure 11B).

#### 3.2.5 All-cause death

Four trials (*n* = 4,223) reported all-cause death (29, 42, 44, 47). Compared with coronary angiography, OCT-guided PCI was not associated with a significant reduction in all-cause death (OR 0.7, 95% CI 0.49–1.02; *p* = 0.550, *I^2^*^ ^= 0%, non-relevant heterogeneity, high certainty, see Figure 10C). Observational studies (52, 54, 55, 57), which included 4 studies with 1,214 patients, also demonstrated that OCT-guided PCI was not significantly associated with a reduction in the risk of all-cause death when compared to coronary angiography (OR 0.87, 95% CI 0.35–2.18; *p* = 0. 211, *I^2^*^ ^= 33.5%, indicating moderate heterogeneity and moderate certainty, as shown in Figure 11C).

#### 3.2.6 MI

Six trials (*n* = 4,747) reported MI (29, 41, 42, 44, 47, 49). Compared with coronary angiography, OCT-guided PCI was not associated with a significant reduction in MI (OR 0.88, 95% CI 0.69 to 1.13; *p* = 0.823, *I^2^* = 0%, non-relevant heterogeneity, high certainty, see Figure 10D). Observational studies (51, 53–57), which included 6 studies with 4,120 patients, also demonstrated that OCT-guided PCI was not significantly associated with a reduction in the risk of MI when compared to coronary angiography (OR 0.66, 95% CI 0.38–1.13; *p* = 0. 319, *I^2^* = 14.9%, indicating low heterogeneity and moderate certainty, as shown in Figure 11D).

#### 3.2.7 TLR

Seven trials (*n* = 4,646) reported TLR (29, 41, 44, 47–50). Compared with coronary angiography, OCT-guided PCI was not associated with a significant reduction in TLR (OR 0.94, 95% CI 0.7–1.27; *p* = 0.856, *I*^2^ = 0%, non-relevant heterogeneity, high certainty, see Figure 10E). However, the results from the observational studies, which included 4 studies with 1,524 patients, showed differing outcomes (51, 52, 54, 57). In contrast to coronary angiography, OCT-guided PCI showed a reduction in the risk of TLR (OR 0.39, 95% CI 0.22–0.68; *p* = 0.384, *I^2^*^ ^= 1.6%, non-relevant heterogeneity, high certainty, see Figure 11E).

#### 3.2.8 TVR

Four trials (*n* = 3,060) reported TVR (29, 42, 47, 50). Compared with coronary angiography, OCT-guided PCI was not associated with a significant reduction in TVR (OR 1.04, 95% CI 0.76–1.43; *p* = 0.857, *I^2^*^ ^= 0%, non-relevant heterogeneity, high certainty, see Figure 10F). Observational studies (53, 54), which included 2 studies with 1,182 patients, also demonstrated that OCT-guided PCI was not significantly associated with a reduction in the risk of TVR when compared to coronary angiography (OR 0.38, 95% CI 0.04–3.37; *p* = 0. 04, *I^2^*^ ^= 76.4%, indicating high heterogeneity and low certainty, as shown in Figure 11F).

#### 3.2.9 Stent thrombosis

Five trials (*n* = 1,886) reported stent thrombosis (43, 44, 46, 48, 49). Compared with coronary angiography, OCT-guided PCI was not associated with a significant reduction in stent thrombosis (OR 0.72, 95% CI 0.38–1.38; *p* = 0.906, *I*^2^ = 0%, non-relevant heterogeneity, high certainty, see Figure 10G). Observational studies (53, 54, 57), which included 3 studies with 1,382 patients, also demonstrated that OCT-guided PCI was not significantly associated with a reduction in the risk of stent thrombosis when compared to coronary angiography (OR 0.61, 95% CI 0.09–4.39; *p* = 0. 101, *I*^2^ = 56.4%, indicating moderate heterogeneity and moderate certainty, as shown in Figure 11G).

## 4 Discussion

OCT, with its high-resolution imaging (10–20 μm), accurately identifies vascular features like thrombi, lipids, and calcium deposits (59–63). In this study, we analyzed 2,758 articles related to OCT and cardiovascular diseases from the WoSCC. In-depth analyses were conducted on these articles by country, institution, journal, author, and keywords using Bibliometrix R software and CiteSpace. This comprehensive exploration revealed the knowledge structure, research hotspots, and emerging trends in the field, laying the groundwork for future strategies in disease prevention and treatment. Our study found that OCT, as a guiding tool for PCI, has become a focal point in recent cohorts and randomized trials, which was further confirmed in our subsequent meta-analysis. After including 11 RCTs and 7 observational studies, we concluded that OCT-guided PCI did not demonstrate significant association with better clinical outcomes. Although the point estimate and the upper bound of the confidence interval hinted at a possible reduction in MACE, cardiac death, all-cause death, MI, TLR, or stent thrombosis with OCT guided PCI, this did not reach statistical significance. However, the meta-analysis of observational studies showed a significant reduction in MACE and TLR.

### 4.1 Advantages and limitations of bibliometric analysis

The United States led in the publication output related to OCT and cardiovascular diseases, also exhibiting the highest proportion of international collaboration. Moreover, China's publication numbers are rapidly growing, likely influenced by recent expert consensus from Chinese cardiology societies emphasizing the importance of OCT in PCI (64). Among the top 10 institutions with the highest publication output, 7 were from the USA, while the remaining were from other countries (China, Singapore, and the UK). Professor Yu Bo from China was the most prolific among the top 10 corresponding authors, followed by authors from the USA. Professor Mehran Roxana held the highest m-INDEX, Professor Yu Bo the largest G-index, and Professor Virmani Renu the highest h-index and total citations. Additionally, among the top 10 most cited papers, one was published in “The Lancet" (37), and three in “Circulation” and other high-impact journals (31, 35, 36). Professor Ik-Kyung Jang's 2005 paper “*in vivo* Characterization of Coronary Atherosclerotic Plaque by Use of Optical Coherence Tomography” ranked highest in citations (31).

Thematic word trend analysis over the past 20 years in the cardiovascular field has centered on eight key terms: coronary artery disease, deep learning, coronary stenosis, heart transplantation, plaque rupture, OCT, congenital heart disease, and cardiovascular diseases. Burst analysis of keywords indicated that studies on OCT and angiography-guided PCI have become hot topics in recent cohorts and randomized trials. Well-developed themes focus on atherosclerotic diseases, blood pressure, stent implantation, and plaque characteristics. The field's attention to the treatment and prognosis of diseases such as “coronary stenosis, acute myocardial infarction, atherosclerosis” post-operation and for survival is also noteworthy.

However, this study has limitations. The primary data for the bibliometric analysis was sourced from the WoSCC. Although the WoSCC includes over 11,000 authoritative and high-impact international academic journals with extensive coverage and powerful analysis features, its singular source may lead to potential article omissions from other databases. Additionally, researchers manually removed papers deemed irrelevant to the study objectives, which might introduce selection bias. Despite these limitations, our study comprehensively analyzes the current state and progress of OCT in cardiovascular research, aiding in identifying future research directions.

### 4.2 Advantages and limitations of meta-analysis

OCT has shown significant technical advantages in the application of cardiovascular diseases (30, 65). Compared to traditional coronary angiography, OCT provides higher-resolution spatial three-dimensional images, critical in accurately assessing plaque composition and morphology. Importantly, OCT optimizes angioplasty of bifurcation lesions, avoiding the common issues of perspective shortening and image overlap in traditional angiography (66). These technical strengths theoretically endow OCT with significant clinical application potential. However, in actual clinical practice, these theoretical advantages of OCT have not entirely translated into clinical benefits. Our meta-analysis of RCTs revealed that OCT-guided PCI did not exhibit significant clinical benefits in MACE, Cardiac death, All-cause death, MI, TLR, TVR, and Stent thrombosis, compared to angiography-guided PCI. Although studies suggest that OCT-guided PCI can achieve a larger minimum lumen diameter (MLD) (53), its use also leads to longer procedural times and higher contrast agent dosages (67), increasing perioperative risks such as early mortality, emergency coronary artery bypass grafting, cancer, and contrast-induced nephropathy (68, 69). These risks might overshadow the clinical benefits of OCT. However, the meta-analysis of observational studies indicated a significant reduction in MACE and TLR with OCT-guided PCI, aligning with previous research (67, 70).

#### 4.2.1 Limitations

When interpreting the results of our meta-analysis, its inherent limitations must be considered. Firstly, the included trials varied in participant populations, outcome definitions, and follow-up periods, potentially affecting comparability and generalizability. Secondly, pre-planned overall and subgroup analyses were conducted at the study level, not at the individual patient level, precluding precise assessment of the specific impact of stent size pre and post PCI guided by OCT on cardiovascular outcomes. Lastly, variations in intravascular imaging guidance standards among different trials could also influence the results.

### 4.3 Comparisons with other studies

Although many meta-analyses have studied intravascular imaging-guided PCI, a systematic review of 24 meta-analyses showed that only 9 focused specifically on RCTs (71). Given the potential introduction of confounding factors in observational studies (71), we conducted separate meta-analyses of evidence from RCTs and observational studies for OCT-guided PCI. This approach differs from previous meta-analyses, showing OCT's significant advantages are more pronounced in observational studies (67, 70), consistent with previous high-quality RCTs (29, 49, 53).

Our results, compared with the study led by Niels R. Holm, showed differences in MACE outcomes (44). The fundamental reason is that calculating OR values directly using incidence rates might differ from results reported in that study, as Cox regression analysis incorporates specific time points of events, often overlooked in simple calculations (44, 72). Additionally, the Cox model typically considers multiple covariates potentially influencing outcomes, such as patient age, gender, and medical history (73). This might be one reason our study did not show a significant clinical advantage of OCT. Furthermore, with a median follow-up time of only 1–2 years in the studies included, detecting statistically significant differences between the two interventions would require longer follow-up and higher event rates.

## 5 Conclusion

In summary, this study primarily employed bibliometric analysis to examine literature published over the past twenty years on OCT and cardiovascular diseases. It identified specific countries, institutions, authors, and journals that have made significant contributions to this field during this period. It was found that OCT as a guiding tool for PCI has become a hot topic in recent cohorts and randomized trials, prompting subsequent meta-analyses. However, OCT-guided PCI did not demonstrate significant clinical benefits, with only the meta-analysis of observational studies suggesting a reduction in MACE and TLR.
