# Supplementary material for: Application of optical coherence tomography in cardiovascular diseases: bibliometric and meta-analysis
Source: Front Cardiovasc Med. 2024 Jul 9;11:1414205. doi: 10.3389/fcvm.2024.1414205 (PMC11263217; doi:10.3389/fcvm.2024.1414205)
Supplement: Supplementary file 1 [file Datasheet1.docx]

## eMethods 1.

Optical Coherence Tomography search terms1 :

TS= ((("Optical Coherence Tomography" OR "Coherence Tomography" OR "OCT")))

Cardiovascular diseases search terms2 :

TS= (((((((((((((((((("coronary heart disease" OR "heart attack" OR "coronary disease" OR" coronary disease" OR "coronary event" OR "congenital heart defect" OR "congenital heart disease" OR "heart valvular disease" OR "aortic valve" OR "mitral valve" OR "tricuspid valve" OR "heart abnormal" OR "cardiovascular diseases"))))))))))))))))))

Search strategy:

TS= ((("Optical Coherence Tomography" OR "Coherence Tomography" OR "OCT"))) AND TS= (((((((((((((((((("coronary heart disease" OR "heart attack" OR "coronary disease" OR" coronary disease" OR "coronary event" OR "congenital heart defect" OR "congenital heart disease" OR "heart valvular disease" OR "aortic valve" OR "mitral valve" OR "tricuspid valve" OR "heart abnormal" OR "cardiovascular diseases"))))))))))))))))))

References

1. Zhang X, Yi K, Xu JG, et al. Application of three-dimensional printing in cardiovascular diseases: a bibliometric analysis. Int J Surg. Published online November 3, 2023.

2. Pan Y, Deng X, Chen X, Lin M. Bibliometric analysis and visualization of research trends in total mesorectal excision in the past twenty years. Int J Surg. Published online September 4, 2023.

## eMethods 2. Literature search.

Working independently and unaware of each other's choices, CC and WJL conducted separate searches of the titles and abstracts of the literature according to the Rayyan platform, a tool designed to support researchers in the independent selection and review of studies. They also manually examined the references of the chosen papers and existing reviews to identify any studies that might have been missed during the initial search. When necessary, authors were contacted for additional information.

We conducted systematic searches of the PubMed, Cochrane Library, Embase, and Web of Science to assemble a comprehensive collection of RCTs and observational studies examining the relationship OCT-guided to angiography-guided PCI in patients with ACS requiring interventional revascularization from the inception of these databases to December 2023. We also searched websites of major cardiovascular and medicine journals (www.nejm.org; https://www.thelancet.com/; https://jamanetwork.com; https://annals.org/aim; https://academic.oup.com/eurheartj; www.onlinejacc.org; and www.ahajournals.org/journal/circ) and bibliographies of relevant studies ^1-4^.Our search strategy incorporated a combination of keywords and Medical Subject Headings (MeSH) searches using the following terms (refer to eTable1 for details). The reference lists of pertinent articles or reviews were also perused to identify additional eligible studies.

To verify potentially relevant studies, the references from retrieved articles and reviews were manually scrutinized. The search strategy comprised a combination of text and Medical Subject Headings (MeSH). Two investigators (CC and WJ L)) independently performed the search. Disagreements between the two authors were resolved by discussion. If the disagreement persisted, another senior investigators (J W) was consulted to attain consensus.

References

1. Buccheri S, Franchina G, Romano S, et al.. Clinical Outcomes Following Intravascular Imaging-Guided Versus Coronary Angiography-Guided Percutaneous Coronary Intervention With Stent Implantation: A Systematic Review and Bayesian Network Meta-Analysis of 31 Studies and 17,882 Patients. JACC Cardiovasc Interv 2017;10:2488-98. 10.1016/j.jcin.2017.08.051.

2. Ahn JM, Kang S-J, Yoon SH, et al.. Meta-analysis of outcomes after intravascular ultrasound-guided versus angiography-guided drug-eluting stent implantation in 26,503 patients enrolled in three randomized trials and 14 observational studies. Am J Cardiol 2014;113:1338-47. 10.1016/j.amjcard.2013.12.043

3. Darmoch F, Alraies MC, Al-Khadra Y, Moussa Pacha H, Pinto DS, Osborn EA. Intravascular Ultrasound Imaging-Guided Versus Coronary Angiography-Guided Percutaneous Coronary Intervention: A Systematic Review and Meta-Analysis. J Am Heart Assoc 2020;9:e013678. 10.1161/JAHA.119.013678

4. Niu Y, Bai N, Ma Y, Zhong PY, Shang YS, Wang ZL. Efficacy of intravascular imaging-guided drug-eluting stent implantation: a systematic review and meta-analysis of randomized clinical trials. BMC Cardiovasc Disord 2022;22:327. 10.1186/s12872-022-02772-w

## eMethods 3. Inclusion Criteria

The criteria for inclusion encompassed all randomized or observational studies investigating the short-term and long-term clinical outcomes of patients who underwent percutaneous coronary intervention (PCI) guided solely by Optical Coherence Tomography (OCT) or angiography. This study encompassed all forms of Acute Coronary Syndrome (ACS), including ST-Elevation Myocardial Infarction (STEMI), Non-ST-Elevation Myocardial Infarction (NSTEMI), and unstable angina pectoris. There were no limitations imposed on the duration of follow-up.

Patients in the experimental group underwent PCI guided by OCT, with or without concurrent angiography. Studies that compared angiography-guided PCI with intravascular imaging (OCT and Intravascular Ultrasound (IVUS)) were considered for inclusion if data specific to the OCT group were available separately.

For observational studies, efforts were made to extract results that accounted for potential confounding factors.

Studies were excluded based on the following criteria: (1) conference abstracts without available full-text articles, (2) failure to report clinical outcomes, (3) lack of appropriate comparison between the groups of interest, (4) comparisons of OCT-guided PCI with methods other than angiography, such as computed tomography or fractional flow reserve, (5) single-group studies, and (6) inability to extract absolute numbers of adverse events from the provided data. The requisite data from eligible studies were extracted and transferred to a pre-specified electronic spreadsheet in Microsoft Excel 2013. General characteristics of the studies were collated, including the first author’s name, country of publication, trial name, study design (observational versus randomized), registration identification number (if applicable), single-center versus multi-center design, participants' baseline cardiac conditions, duration of follow-up, average age of participants, percentage of male participants, and data on potential comorbidities of the participants, including hypertension, dyslipidemia, diabetes, and current smoking status. Specific data extracted for synthesis included the sample size of the study group and the incidence rate of adverse clinical outcomes.

Double publications, case reports, case series without control groups, reviews, and conference abstracts were not included in the analysis. Studies that solely employed conservative treatments, such as guideline-directed medical therapy (GDMT), as a comparator to OCT-guided PCI, were deemed ineligible for analysis.

## eMethods 4. Data extraction

The data extraction methodology for our study was meticulously designed to gather comprehensive information from each selected study. This process involved the collation of key attributes from the studies as well as detailed baseline characteristics of the patients within the two groups: those undergoing Optical Coherence Tomography (OCT)-guided percutaneous coronary intervention (PCI) and those receiving PCI guided by angiography.

Specifically, the data extraction protocol encompassed the following elements:

1. Study Characteristics: This included the extraction of general features of each study, such as the name of the first author, the country of publication, the title of the trial, and the study design, distinguishing between observational and randomized approaches. Where applicable, the registration identification number was also recorded.

2. Location and Time Period Under Observation: We noted the geographical location of each study and the time frame during which the observation was conducted.

3. Study Type and Comparator Treatment: The type of study (whether it was single-center or multi-center) and the comparator treatment used in each study were documented.

4. Follow-up Period: The median duration of follow-up for the study participants was noted.

5. Baseline Characteristics of Patients: For both the OCT and Angiography groups, we extracted detailed baseline patient data. This included the median age of the patients, the percentage of male patients, and the prevalence of potential comorbidities such as hypertension, diabetes, smoking habits, and dyslipidemia.

6. Type of Acute Coronary Syndrome and Management: We gathered data on the types of acute coronary syndrome (ACS) present in each group, specifically detailing the incidence of unstable angina, NSTEMI (Non-ST-Elevation Myocardial Infarction), and STEMI (ST-Elevation Myocardial Infarction), along with their respective management strategies.

Through this comprehensive data extraction process, we aimed to provide a robust and detailed analysis of the clinical outcomes and characteristics of patients undergoing OCT-guided versus angiography-guided PCI. This methodological approach ensures a thorough evaluation of the available literature, contributing to a more informed understanding of the comparative effectiveness of these two diagnostic modalities in the management of acute coronary syndromes.

## eMethods 5. Quality assessment

Risk of bias appraisal was performed using two tools. For RCTs, the Cochrane tool for assessing the risk of bias in randomized trials (RoB2 tool) was used ^1^.

This tool evaluates the quality of an RCT in five main domains including the randomization process, deviations from the intended interventions, missing outcome data, measurement of the outcome, and selection of the reported results.By the end we give an overall rating. Each of the domains and the overall judgment is either rated as low, with some concerns,or a high risk of bias. The overall judgment is rated as low if all the domains are judged to be at low risk and some concerns if two or fewer domains are rated as some concerns but none of them are at high risk. A study was rated as high risk of bias if at least one area was judged to be at high risk or three or more domains were judged as some concerns.

For observational studies, the Newcastle-Ottawa Quality Assessment Form for Cohort Studies was filled out for the eligible studies. This tool assesses each study based on three main domains of selection, comparability, and outcome and rates the studies based on the stars given to each study during quality assessment. Each study is rated as good, fair, and poor based on the pre-specified criteria.

References

1. Sterne JAC, Savovi´c J, Page MJ, et al. RoB 2: a revised tool for assessing risk of bias in randomised trials. BMJ. 2019;366:l4898 (Clinical research ed).

## eTable 1 The search strategy

| Databases | Search strategy |
| --- | --- |
| PubMed | ("OCT"[All Fields] OR ("tomography, optical coherence"[MeSH Terms] OR ("tomography"[All Fields] AND "optical"[All Fields] AND "coherence"[All Fields]) OR "optical coherence tomography"[All Fields] OR ("optical"[All Fields] AND "coherence"[All Fields] AND "tomography"[All Fields]))) AND ("angiography"[MeSH Terms] OR "angiography"[All Fields] OR "angiographies"[All Fields] OR "angiography s"[All Fields]) AND ("percutaneous coronary intervention"[MeSH Terms] OR ("percutaneous"[All Fields] AND "coronary"[All Fields] AND "intervention"[All Fields]) OR "percutaneous coronary intervention"[All Fields]) |
| Embase | ('optical coherence tomography'/exp OR 'optical coherence tomography' OR (('optical'/exp OR optical) AND ('coherence'/exp OR coherence) AND ('tomography'/exp OR tomography))) AND angiography:ti,ab,kw AND 'percutaneous coronary intervention':ti,ab,kw |
| Cochrane Library | #1angiography (Word variations have been searched)  #2[mh angiography] (Word variations have been searched)  #3PCI (Word variations have been searched)  #4percutaneous coronary intervention (Word variations have been searched)  #5[mh "percutaneous coronary intervention"] (Word variations have been searched)  #6OCT (Word variations have been searched)  #7optical coherence tomography  #8[mh "optical coherence tomography"] (Word variations have been searched)  #9 #1 OR #2  #10 #3 OR #4 OR #5  #11 #6 OR #7 OR #8  #12 #9AND #10 AND #11 |

## eFigure 1. Quality Assessment of 11 RCT Studies


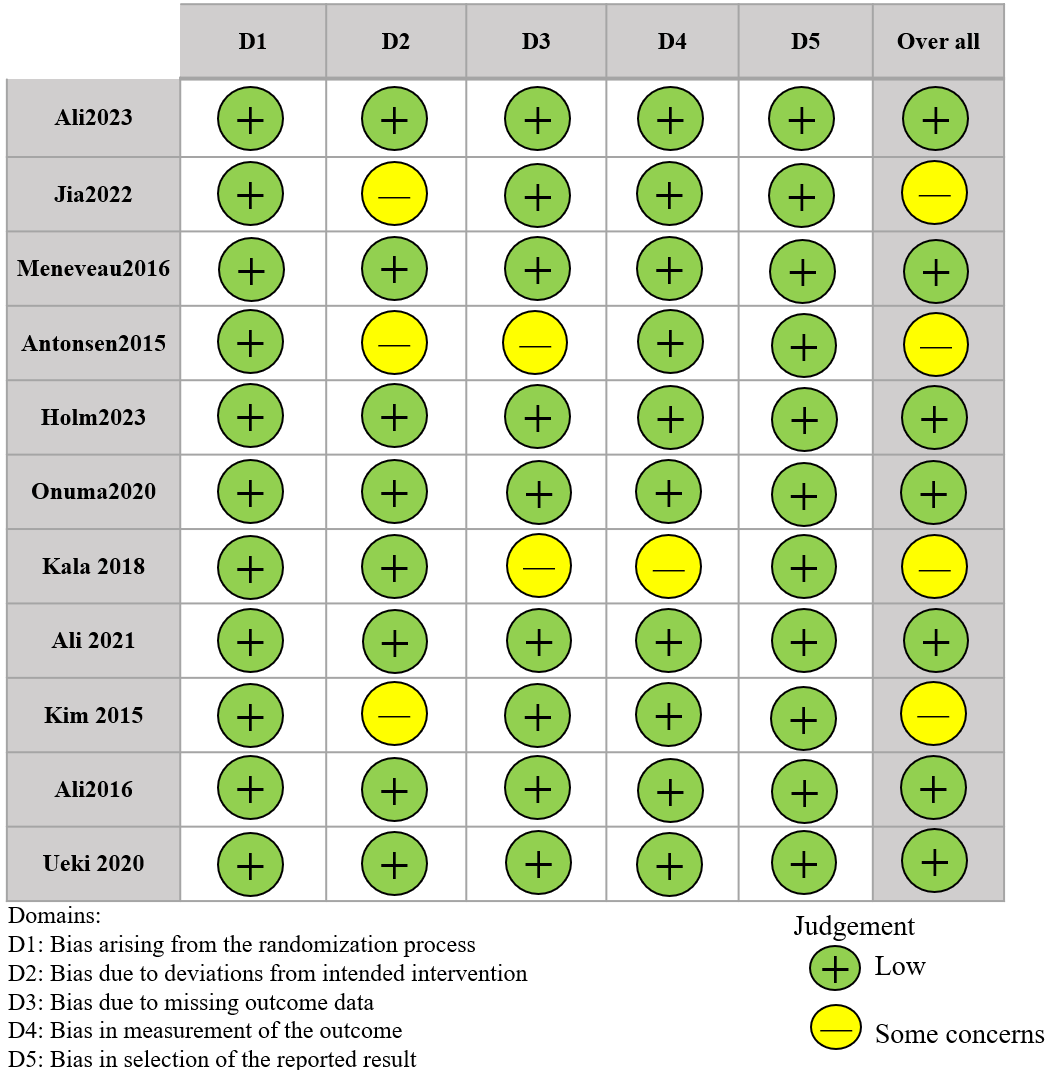


## eTable 2 Quality assessment of the included studies

| Study (Observational) | Representativeness | Selection of the nonexposed | Ascertainment of exposure | Demonstration of nonpresence of outcome at the start | Comparability (analysis controlled for confounders) | Assessment | Duration of follow-up | Adequacy of follow-up | Overall Quality |
| --- | --- | --- | --- | --- | --- | --- | --- | --- | --- |
| Khalifa 2021 | ★ | ★ | ★ | ★ |  | ★ | ★ | ★ | Poor |
| D'Ascenzo 2017 | ★ | ★ | ★ | ★ | ★ | ★ | ★ | ★ | Poor |
| Sheth 2016 | ★ | ★ | ★ | ★ | ★★ | ★ | ★ | ★ | Good |
| Iannaccone 2016 | ★ | ★ | ★ | ★ | ★★ | ★ | ★ | ★ | Good |
| Antonsen 2015 | ★ | ★ | ★ | ★ |  | ★ | ★ | ★ | Poor |
| Di Giorgio 2013 | ★ | ★ | ★ |  |  | ★ | ★ | ★ | Poor |
| Cortese 2021 | ★ | ★ | ★ | ★ | ★★ | ★ | ★ | ★ | Good |
| Okura 2018 | ★ | ★ | ★ | ★ |  | ★ | ★ | ★ | Poor |
| Jones 2018 | ★ | ★ | ★ | ★ | ★★ | ★ | ★ | ★ | Good |

| **eTable 2** | | | | | |  | | | | | | |  | | |
| --- | --- | --- | --- | --- | --- | --- | --- | --- | --- | --- | --- | --- | --- | --- | --- |
| Study characteristics | | | | | | Baseline characteristics of patients included (OCT group/Angiography group) | | | | | | | Type of acute coronary syndrome and management (OCT group/Angiography group) | | |
| Author Year | Location | Time Period  Under Observation | Study type | Comparator treatment | Follow-up period (median) | Patients | Age,  median | Male  patients | Hypertension | Diabetes | Smoking | Dyslipidemia | Unstable  Angina | NSTEMI | STEMI |
| 3 Khalifa 2021 | Multinational | 2009-2018 | Observational | Angiography | 1 year | 260/130 | 70 (13)/73 (11) | 176 (68)/90 (69) | 219 (84)/109 (84) | 101 (39)/61 (47) | 78 (30)/50 (38) | 196 (75)/97 (75) | 18(7)/15 (12) | 42(16)/18 (14) | 200(77)/97 (75) |
| 4 D'Ascenzo 2017 | Multinational | 2009-2012,2014-2015 | Observational | Angiography  FFR guid-  ance | 25 months | 197/197 | 63 (13)/64 (10) | 153 (77.7)/151 (76.6) | 133 (67.5)/139 (70.6) | 38 (19.3)/48 (24.4) | 113 (57.4)/120 (60) | 114 (57.9)/109 (55.3) | 54(27.4)/53 (26.9) | 89 (45.2)/80 (40.9) | 54(27.4)/65 (33) |
| 5 Sheth 2016 | Multinational | 2010-2014 | Observational | Angiography | 12 months | 214/428 | 60.9 (11.5)/61.2 (12.1) | 167 (78)/354 (82.7) | - | 38 (17.8)/79 (18.5) | 93 (43.5)/184 (43.0) | - | 0/0 | 0/0 | 214(100)/428(100) |
| 9 Di Giorgio 2013 | Italy | 2009-2010 | Observational | Angiography | 12 months | 40/40 | 67.9 (11.3)/62.7 (10.9) | 35 (87.5)/31 (77.5) | 30 (75)/32 (80) | 12 (30)/13 (32.5) | 18 (45)/22 (55) | 29 (72.5)/19 (47.5) | 0/0 | 16(40)/21 (52.5) | 24(60)/19 (47.5) |
| 7 Iannaccone 2016 | Multinational | 2009-2015,2014-2015 | Observational | Angiography | 700 days | 270/270 | 60 (13)/  61 (12) | 226 (79)/227 (79) | 160 (56)/168 (59) | 49 (17)/48 (18) | 162 (57)/163 (58) | 142 (49)/135 (47) | 46(16)/42 (15) | 99(34)/93 (36) | 140(49)/150 (53) |
| 18 Jones 2018 | United Kingdom | 2005-2015 | Observational | Angiography | 4.8 years | 1149/75046 | 62.7(12.1)/65.2(11.7) | 794(69.1)/55,979 (74.1) | 649 (56.5)/39,737 (52.6) | 288 (27.5)/17,488 (12.1) | 488 (42.5)/37,292 (49.3) | 643 (55.9)/41,048 (45.3) | - | -- | - |
| 19 Cortese 2021 | Multinational | 2012-2017 | Observational | Angiography | 12 months | 162/353 | 69 (10)/71(10) | 120 (74.3)/272 (76.7) | 134 (83.1)/258 (73.4) | 26 (16.7)/120 (34.3) | 63 (39.2)/187 (53.4) | 112 (68.9)/170 (48.2) | 49 (30.8)/99 (27.7) | 44 (27.4)/85 (24.3) | 16 (9.6)/42 (12.3) |

**1. Ali 2023 Optical Coherence Tomography–Guided versus Angiography-Guided PCI**

**Data Analysis for Specific Lesions (Left Main, Non-Left Main, Long Lesions, Calcified, etc.):**

The study does not explicitly mention an analysis that segregates outcomes by specific lesion characteristics such as left main, non-left main, long lesions, or calcification within the coronary arteries. Typically, in clinical trials like the one described, data regarding lesion-specific outcomes would be either part of a subgroup analysis or detailed in the supplementary materials if they significantly impact the primary or secondary endpoints. For this particular trial, the primary focus was on comparing OCT-guided PCI to angiography-guided PCI regarding minimum stent area and target-vessel failure.

**Differentiation of ACS vs. Non-ACS Patients:**

The trial included patients with varying clinical presentations, which likely include both acute coronary syndrome (ACS) and non-ACS patients. Patients were enrolled based on the presence of myocardial ischemia, high-risk patient features (like medication-treated diabetes), or high-risk lesion characteristics. The study mentioned categorizing patients by clinical presentation (e.g., silent ischemia, stable angina, unstable angina, non-STEMI, recent STEMI). However, it does not specify a separate analysis or outcomes stratified explicitly by ACS versus non-ACS statuses.

**2. Holm 2023：OCT or Angiography Guidance for PCI in Complex Bifurcation Lesions**

**Data Involving Specific Lesion Types (Left Main, Non-Left Main, Long Lesions, Calcified, etc.):**

The study specifically mentioned lesions at the left main coronary artery bifurcation. Among the 1201 patients, 227 (18.9%) had a lesion located at a left main coronary-artery bifurcation. This subgroup was directly compared in terms of outcomes between the OCT-guided PCI and angiography-guided PCI groups.

The OCTOBER trial also conducted an analysis that differentiated patients by the complexity of lesions, which included long and calcified lesions, using a stratification method that included severity of calcification (none-to-minor vs. moderate-to-severe).

**Distinction between ACS and non-ACS Patients, and the Effectiveness of OCT in These Groups:**

The trial differentiated between patients with ACS (Acute Coronary Syndrome) and non-ACS. Specifically, the patient population included those with stable angina, unstable angina, or non-ST-segment elevation myocardial infarction. This distinction allowed for subgroup analyses to examine the outcomes of OCT-guided PCI in these different patient categories.

Outcomes were assessed in terms of major adverse cardiac events (MACE), and the subgroup analyses explored whether ACS versus non-ACS status affected the benefits of OCT-guided PCI.

**3. Jia 2022：EROSION III A Multicenter RCT of OCT-Guided Reperfusion in STEMI With Early Infarct Artery Patency**

**Data Involving Specific Lesion Types (Left Main, Non-Left Main, Long Lesions, Calcified, etc.):**

The study included patients with STEMI who presented with angiographic diameter stenosis ≤ 70% and Thrombolysis In Myocardial Infarction (TIMI) flow grade 3, indicating a specific patient subset with early patency of the infarct artery. The study focused on these less severe occlusions but did not explicitly break down data by lesion types like left main, non-left main, long lesions, or calcified lesions beyond the initial stenosis percentage.

**Distinction between ACS and non-ACS Patients, and the Effectiveness of OCT in These Groups:**

The EROSION III trial specifically enrolled patients with acute coronary syndrome (ACS), particularly those with STEMI. Therefore, all participants in this study had ACS, and there was no subgroup of non-ACS patients. The study’s focus was to optimize reperfusion strategies in this acute setting using OCT guidance to potentially reduce the need for stent implantation while achieving favorable outcomes.

The primary efficacy endpoint was the rate of stent implantation, with findings showing a significant reduction in stent usage in the OCT-guided group compared to the angiography-guided group (43.8% vs. 58.8%, P = 0.024). This indicates OCT's potential to more accurately assess the necessity for stenting in acute settings.

**4. Ali 2021：Optical Coherence Tomography–Guided versus Angiography-Guided PCI**

**Data Involving Specific Lesion Types (Left Main, Non-Left Main, Long Lesions, Calcified, etc.):**

The study included patients with various high-risk coronary-artery lesions. These included lesions responsible for recent myocardial infarction, long or multiple lesions requiring more than 28 mm of stent, bifurcation lesions requiring two stents, severely calcified lesions, chronic total occlusions, and diffuse or multifocal in-stent restenosis.

This comprehensive inclusion of different lesion types allowed the study to assess OCT-guided PCI across a spectrum of complex cases typically challenging to manage with standard angiography alone.

**Distinction between ACS and non-ACS Patients, and the Effectiveness of OCT in These Groups:**

All patients in this study were considered high-risk due to either their diabetes status or the complexity of their coronary lesions. However, the study particularly included patients who had lesions responsible for a recent myocardial infarction, indicating a subset with ACS.

The trial did not explicitly separate the outcomes between ACS and non-ACS groups in its primary analysis. However, it did stratify randomization based on whether patients presented with a myocardial infarction, thus indirectly considering the impact of ACS in its procedural and clinical outcome evaluation.

**5. Onuma 2020：A Randomized Trial Evaluating Online 3-Dimensional Optical Frequency Domain Imaging–Guided Percutaneous Coronary Intervention in Bifurcation Lesions**

**Data Involving Specific Lesion Types (Left Main, Non-Left Main, Long Lesions, Calcified, etc.):**

The study specifically investigated bifurcation lesions treated with a provisional single stent strategy using the Ultimaster sirolimus-eluting stent. It focused on improving outcomes by using online 3-dimensional optical frequency domain imaging (3D-OFDI) to guide stent placement and reduce incomplete stent apposition at bifurcation points. The lesions included were primarily bifurcation involving the main vessel and a significant side branch, detailed as Medina classifications in the study. This included configurations where both main and side branches were diseased or only one was, covering a variety of bifurcation complexities. However, there's no explicit breakdown in the study results by lesion types such as left main or non-left main, or based on the lesion characteristics like length or calcification.

**Distinction between ACS and non-ACS Patients, and the Effectiveness of OCT in These Groups:**

The study does not explicitly categorize or analyze the data based on acute coronary syndrome (ACS) versus non-ACS conditions. The inclusion criteria were focused on anatomical characteristics of the bifurcation lesions rather than the clinical presentation of ACS. Therefore, it does not provide a direct analysis or comparison of OCT outcomes specifically between ACS and non-ACS patient groups. Most patients presented with stable angina or silent ischemia, indicating a non-acute setting for the interventions.

**6. Ueki 2020 ：Randomized comparison of optical coherence tomography versus angiography to guide Bioresorbable vascular scaffold implantation: The OPTICO BVS study**

**Data Involving Specific Lesion Types (Left Main, Non-Left Main, Long Lesions, Calcified, etc.):**

The studt do not specifically analyze the effects of OCT guidance on different types of lesions such as left main, non-left main, long lesions, or calcified lesions separately. The focus was more generally on the effectiveness of OCT versus angiography guidance during bioresorbable vascular scaffold implantation.

**Distinction between ACS and non-ACS Patients, and the Effectiveness of OCT in These Groups:**

the study distinguishes between ACS and non-ACS patients. It evaluates how OCT guidance influences outcomes in these different groups by examining variables like scaffold expansion and malapposition. However, due to early termination of the study and limited sample size, conclusive results were not achieved​

**7. Kala 2017: OCT guidance during stent implantation in primary PCI: A randomized multicenter study with nine months of optical coherence tomography follow-up**

**Data Involving Specific Lesion Types (Left Main, Non-Left Main, Long Lesions, Calcified, etc.):**

The study does not specifically address different lesion types such as left main, non-left main, long lesions, or calcified lesions. The focus is primarily on the overall impact of OCT guidance during primary PCI in patients with STEMI.

**Distinction between ACS and non-ACS Patients, and the Effectiveness of OCT in These Groups:**

This study specifically involves patients with acute coronary syndrome (ACS), particularly those with ST-elevation myocardial infarction (STEMI), undergoing primary PCI. It does not compare ACS with non-ACS patients but rather examines the outcomes of OCT-guided PCI versus angiography-guided PCI in this specific patient group.

**8. Ali 2016: Optical coherence tomography compared with intravascular ultrasound and with angiography to guide coronary stent implantation (ILUMIEN III: OPTIMIZE PCI): a randomised controlled trial**

**Data Involving Specific Lesion Types (Left Main, Non-Left Main, Long Lesions, Calcified, etc.):**

OPTIMIZE PCI study compares OCT, IVUS, and angiography for coronary stent implantation guidance. It excludes specific lesion types such as left main or ostial right coronary artery stenoses, chronic total occlusions, and others, focusing on lesions in native coronary arteries that are less than 40 mm long and with a diameter between 2.25 and 3.50 mm.

**Distinction between ACS and non-ACS Patients, and the Effectiveness of OCT in These Groups:**

The study does not specifically categorize or analyze patients based on ACS versus non-ACS conditions but focuses on general outcomes and procedural safety across the different imaging guidance methods used.

**9. Meneveau 2016: Optical Coherence Tomography to Optimize Results of Percutaneous Coronary Intervention in Patients with Non–ST-Elevation Acute Coronary Syndrome Results of the Multicenter, Randomized DOCTORS Study (Does Optical C oherence Tomography Optimize Results of Stenting)**

The study focused on the use of optical coherence tomography (OCT) in optimizing percutaneous coronary intervention (PCI) for patients with non-ST-segment elevation acute coronary syndromes (NSTE-ACS). It compared OCT-guided PCI to fluoroscopy-guided PCI. OCT use led to changes in procedural strategy in 50% of the cases in the OCT group and achieved higher post-PCI fractional flow reserve values. There were no significant differences in myocardial infarction rates or procedural complications between the two groups.

**10. Kim 2015: Randomized Comparison of Stent Strut Coverage Following Angiography- or Optical Coherence Tomography-guided Percutaneous Coronary Intervention**

The study compared angiography-guided versus optical coherence tomography (OCT)-guided percutaneous coronary interventions (PCIs) in terms of stent strut coverage. A total of 101 patients with 105 lesions were enrolled, either under OCT guidance (51 lesions) or angiography guidance (54 lesions). Six months post-intervention, the OCT-guided group showed significantly lower percentages of uncovered and malapposed stents. This suggests that OCT guidance may enhance stent strut coverage compared to angiography alone, potentially improving long-term outcomes.

**11. Antonsen 2015: Optical Coherence Tomography Guided Percutaneous Coronary Intervention With Nobori Stent Implantation in Patients With Non–ST-Segment–Elevation Myocardial Infarction (OCTACS) Trial Difference in Strut Coverage and Dynamic Malapposition Patterns at 6 Months**

**Data Involving Specific Lesion Types (Left Main, Non-Left Main, Long Lesions, Calcified, etc.):**

The study does not explicitly categorize or analyze data based on specific lesion types such as left main, non-left main, long lesions, or calcified lesions. It focuses on overall outcomes of OCT-guided versus angiography-guided stent implantation without specific breakdowns by lesion type.

**Distinction between ACS and non-ACS Patients, and the Effectiveness of OCT in These Groups:**

The study does not distinguish between ACS and non-ACS patients in the dataset. It broadly addresses the effects of OCT on stent strut coverage in a general patient population undergoing drug-eluting stent implantation, without specifically segmenting the analysis by these patient categories.
